# Molecular diversity of mitochondrial autophagy receptors: context-dependent effects in human health and disease

**DOI:** 10.1038/s41420-026-03207-7

**Published:** 2026-06-25

**Authors:** Ana Rožić, Mija Marinković, Ivana Novak

**Affiliations:** 1 School of Medicine, University of Split, Split, Croatia; 2 Faculty of Science, University of Split, Split, Croatia

**Keywords:** Mitophagy, Mitochondrial proteins, Phosphorylation, Cancer

## Abstract

Mitophagy receptors are central regulators of mitochondrial quality control, integrating metabolic, stress-related, and developmental cues to maintain cellular homeostasis. Accumulating evidence indicates that their dysregulation contributes to a broad spectrum of human diseases through highly context-dependent mechanisms. In cardiovascular and neurological disorders, receptor-mediated mitophagy shapes cell survival, synaptic function, stress adaptation, and tissue integrity, with both insufficient and excessive activity proving detrimental. In cancer, mitophagy receptors display dual and stage-specific roles, acting as tumor suppressors in early disease while later supporting metabolic adaptation, stemness, and therapy resistance. Metabolic diseases highlight the tissue-specific complexity of mitophagy regulation, where precise control of mitochondrial turnover is essential for insulin sensitivity, calcium signaling, and energy homeostasis. In hematological, inflammatory, and autoimmune disorders, receptor-mediated mitophagy emerges as a fundamental determinant of lineage commitment, immune cell function, and inflammatory balance. Collectively, these findings position mitophagy receptors not as uniform stress responders, but as dynamic modulators of disease progression, whose precise and context-sensitive targeting may offer novel therapeutic opportunities across diverse pathological conditions.

## Facts

- Receptor-mediated mitophagy constitutes a specialized mitochondrial quality control system that integrates mitochondrial turnover with apoptosis, metabolism, and cell fate regulation.
- Individual mitophagy receptors function in a strictly context- and tissue-dependent manner, allowing the same pathway to confer either cytoprotection or vulnerability.
- Pathological states arise from qualitative dysregulation of receptor-specific mitophagy rather than from global autophagy failure, indicating a narrow homeostatic window for mitochondrial clearance.
- Effective therapeutic targeting of mitophagy will require receptor-selective and temporally restricted modulation to preserve physiological mitochondrial homeostasis.

## Open Questions

- Why do mitophagy receptors exert distinct effects across diseases and tissues?
- Can receptor-selective and temporally controlled modulation of mitophagy be achieved therapeutically?
- What are the long-term consequences of modulating individual mitophagy receptors on tissue homeostasis and disease progression?
- Can disease-associated alterations in mitochondrial structure or metabolism predict dependence on specific mitophagy receptors?

## Introduction

Mitochondrial quality control is essential for maintaining cellular energy balance, redox homeostasis, and survival under stress [1]. Mitophagy, a selective form of autophagy that removes damaged or surplus mitochondria, represents a critical component of this regulation. Mechanistically, mitophagy proceeds through two principal routes: ubiquitin-dependent and receptor-mediated pathways [2].

In the canonical ubiquitin-dependent pathway, mitochondrial depolarization stabilizes PTEN-induced putative kinase 1 (PINK1) on the outer mitochondrial membrane (OMM), leading to recruitment of the E3 ubiquitin ligase Parkin and extensive ubiquitination of mitochondrial surface proteins. These ubiquitin chains are recognized by adaptor proteins: sequestosome 1 (SQSTM1, hereafter referred to as p62), NBR1, optineurin (OPTN), Tax1-binding protein 1 (TAX1BP1), and calcium-binding and coiled-coil domain 2 (CALCOCO2, hereafter referred to as NDP52), which contain both ubiquitin-binding domains and LC3-interacting region (LIR) motifs, thereby linking damaged mitochondria to microtubule-associated protein 1 light chain 3/gamma-aminobutyric acid receptor-associated protein (LC3/GABARAP)-positive autophagosomes. In addition to their role as ubiquitin-binding adaptors, these soluble receptors also contribute to autophagosome initiation [3].

Beyond PINK1-Parkin signaling, ubiquitin-independent mechanisms rely on mitochondria-anchored receptors that directly recruit the autophagic machinery. These receptors are embedded in mitochondrial membranes and act as molecular bridges between mitochondria and autophagosomes. Among the first identified were BNIP3 (BCL2/adenovirus E1B 19 kDa protein-interacting protein 3) and its homolog BNIP3L/NIX (BCL2/adenovirus E1B 19 kDa protein-interacting protein 3-like), BCL-2 homology 3 (BH3)-only proteins that function dually as pro-apoptotic factors and mitophagy receptors [4–6]. These receptors form stable homodimers that can enhance mitophagy [4, 7], although transmembrane-mediated dimerization is not strictly required when membrane anchoring is achieved through alternative mechanisms [8]. Both engage LC3/GABARAP proteins via a conserved LIR motif. Phosphorylation of serine residues flanking the LIR motif enhances receptor–autophagosome binding and mitophagy efficiency [9, 10]. BNIP3- and BNIP3L/NIX-mediated mitophagy has been linked to recruitment of early autophagy machinery via phosphoinositide-interacting protein 2 (WIPI2) [8, 11], while recent evidence suggests additional roles in isolation membrane tethering and expansion [12]. BNIP3 and BNIP3L/NIX receptor abundance is tightly regulated by ubiquitin-dependent turnover mediated by the SKP1-Cul1-F-box and leucine-rich repeat protein 4 (SCF-FBXL4) E3 ubiquitin ligase [13–15], and by the mitochondrial protein phosphatase targeting COQ7 (PPTC7), which modulates their phosphorylation and stability [16–18]. In addition, AMP-activated protein kinase (AMPK) imposes pathway selectivity by suppressing BNIP3- and BNIP3L/NIX-dependent mitophagy while promoting PINK1-Parkin-mediated mitochondrial clearance [19].

The existence of additional OMM receptors further diversifies, emphasizing the complexity of mitophagy regulation. In particular, FUNDC1 (FUN14 domain-containing protein 1) integrates mitochondrial dynamics and mitophagy through phosphorylation-dependent regulation of LC3 binding. FUNDC1 coordinates mitochondrial fission by interacting with dynamin-related protein 1 (DRP1) and optic atrophy 1 (OPA1), thereby functionally coupling mitochondrial dynamics to mitophagy [20]. Its activity is tightly controlled by reversible phosphorylation, where unc-51-like autophagy-activating kinase 1 (ULK1) promotes mitophagy through activating phosphorylation of FUNDC1, whereas Src proto-oncogene tyrosine kinase (Src) and casein kinase 2 (CK2) inhibit LC3 binding. In contrast, the mitochondrial phosphatase phosphoglycerate mutase family member 5 (PGAM5) restores FUNDC1–LC3 interaction through dephosphorylation, thereby promoting mitophagy [21]. A functional mammalian homolog of yeast Atg32, BCL2L13 (BCL2-like 13), promotes stress-induced mitochondrial clearance. Its activity is regulated by AMP-activated protein kinase catalytic subunit alpha 2 (AMPKα2)-dependent phosphorylation, which modulates LC3/GABARAP engagement and receptor function [22, 23]. Furthermore, FKBP8 (FK506-binding protein 8) OMM protein exerts anti-apoptotic effects through interactions with B-cell lymphoma 2/B-cell lymphoma-extra large (BCL-2/BCL-XL) [24]. In mitophagy, FKBP8 functions as an LC3-binding receptor that facilitates mitochondrial fragmentation and clearance. It also supports early autophagosome formation during starvation via its membrane-anchoring domain, underscoring stimulus-dependent functional versatility [25, 26]. AMBRA1 (activating molecule in Beclin1-regulated autophagy protein 1) serves as an example of a mitophagy receptor that supports both Parkin-dependent and -independent pathways. During Parkin-independent mitophagy, phosphorylation of AMBRA1 by inhibitory kappa B kinase α (IKKα) stabilizes its interaction with LC3, thereby enhancing mitophagic clearance [27].

**Fig. 1:**
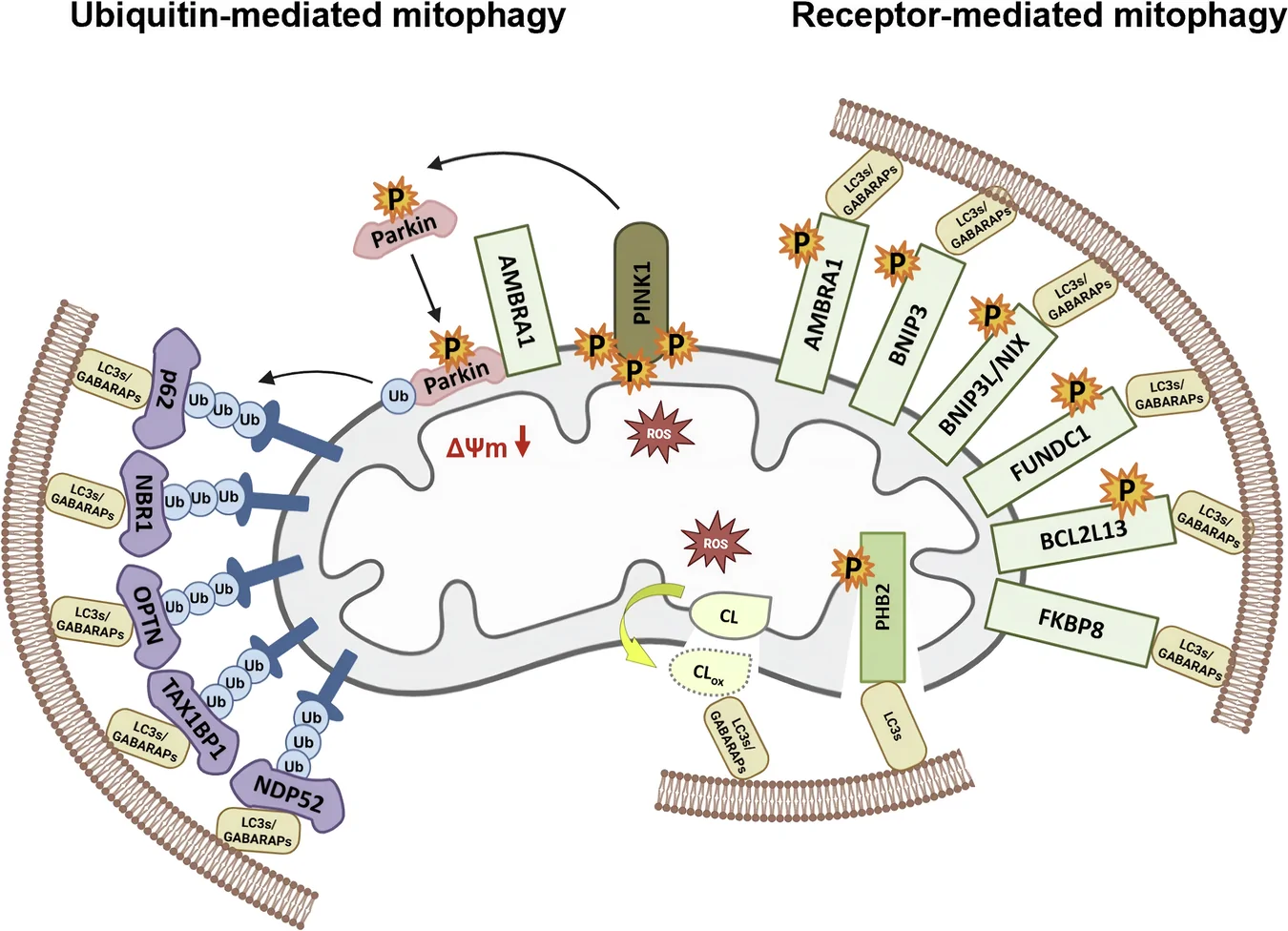
Ubiquitin-dependent and receptor-mediated pathways of mitophagy. Schematic overview of the two principal mechanisms regulating selective mitochondrial clearance. In the ubiquitin-dependent pathway, mitochondrial depolarization stabilizes PINK1 on the OMM, leading to Parkin activation and ubiquitination of mitochondrial surface proteins. Ubiquitin chains are recognized by adaptor proteins (p62/SQSTM1, NBR1, OPTN, TAX1BP1, NDP52), which link damaged mitochondria to LC3/GABARAP-positive phagophores. In receptor-mediated mitophagy, mitochondria-anchored receptors (BNIP3, BNIP3L/NIX, FUNDC1, BCL2L13, FKBP8, AMBRA1) directly recruit LC3/GABARAP via LIR motifs, frequently regulated by phosphorylation. Notably, AMBRA1 can function in both Parkin-dependent and Parkin-independent contexts. At the IMM, PHB2, and cardiolipin exposed upon mitochondrial stress also promote LC3 engagement. Abbreviations: CL cardiolipin, CL_ox_ oxidized cardiolipin, IMM inner mitochondrial membrane, LIR LC3-interacting region, OMM outer mitochondrial membrane, P phosphorylation, ROS reactive oxygen species, Ub ubiquitin, ΔΨm mitochondrial membrane potential.

In addition to OMM receptors, components of the inner mitochondrial membrane (IMM) have emerged as important regulators of mitophagy. The first one identified, PHB2 (prohibitin 2) recruits autophagosomes through its Tyr–Gln–Arg–Leu LIR motif, facilitating the removal of damaged mitochondria during Parkin-mediated mitophagy and paternal mitochondrial clearance in *C. elegans* [28, 29]. Surprisingly, Anton et al. identified cardiolipin as an atypical inducer of autophagy. Although normally confined to the IMM, cardiolipin can redistribute to the OMM under stress, where it directly interacts with LC3/GABARAP proteins to facilitate mitochondrial clearance [30]. The major outer and inner mitochondrial membrane mitophagy receptors and their regulatory mechanisms are schematically summarized (Fig. 1). Collectively, these findings highlight that mitophagy receptor function is tightly controlled by post-translational modifications, including phosphorylation, ubiquitination, and other regulatory modifications such as acetylation, glycosylation and SUMOylation, which fine-tune receptor stability, protein–protein interactions, and pathway selectivity [31].

Although mitophagy receptors share structural features such as transmembrane anchoring and LIR motifs, they differ in activation cues and regulatory control. While soluble ubiquitin-binding adaptors and the PINK1-Parkin axis are central to canonical damage-induced mitophagy, they operate within a distinct ubiquitin-centered cascade. In this review, we focus on mitochondria-anchored receptors, BNIP3, BNIP3L/NIX, FUNDC1, BCL2L13, AMBRA1, FKBP8, PHB2, and cardiolipin, and examine their context-dependent roles in human health and disease.

## Cardiovascular diseases

Cardiac hypertrophy is an adaptive remodeling response to chronic pressure or volume overload that serves to normalize ventricular wall stress but, under sustained stress, frequently progresses to maladaptive remodeling and heart failure driven by cardiomyocyte loss and apoptosis [32, 33]. Both BNIP3L/NIX and its homolog BNIP3 mediate stress-induced cardiomyocyte apoptosis, with low expression in normal hearts [34–36]. BNIP3 is induced by hypoxia via hypoxia-inducible factor-dependent pathways, whereas BNIP3L/NIX is upregulated in response to hypertrophy, defining distinct apoptotic pathways [34, 37]. Overexpression of BNIP3L/NIX alone has minimal apoptotic effect in adult hearts but can exacerbate apoptosis under hypertrophic signaling, especially in neonatal models [38]. Beyond apoptosis, both receptors help regulate mitochondrial quality in cardiomyocytes through “mitochondrial pruning”, limiting mitochondrial overproliferation and protecting against age-related cardiomyopathy [39]. In contrast, FUNDC1-dependent mitophagy protects against hypertrophy, as remodeling aldehyde dehydrogenase 2 (ALDH2) activation restores FUNDC1 expression via nuclear respiratory factor 1 (NRF1)-dependent transcriptional control, thereby limiting maladaptive remodeling [40].

In hypertensive cardiac hypertrophy, mitophagy plays a dual role, as both insufficient and excessive activation promote mitochondrial dysfunction and disease progression. BNIP3/NIX-mediated mitophagy exemplifies this duality, supporting mitochondrial quality control at moderate levels but promoting apoptosis when overactivated. In contrast, FUNDC1-mediated mitophagy is generally cardioprotective, and its loss exacerbates mitochondrial damage and cardiac dysfunction [41]. More broadly, emerging evidence indicates that mitochondrial dysfunction in hypertension is accompanied by increased autophagic activity, potentially as a compensatory response to oxidative stress, while the role of mitophagy has not been specifically investigated [42].

In addition to hypertrophic and ischemic injury, BNIP3 and FUNDC1 have opposing roles in doxorubicin (DOX)-induced cardiotoxicity. While BNIP3 promotes mitochondrial dysfunction, ROS production, and necrotic death of cardiomyocytes [43], FUNDC1 exerts cardioprotective effects by preserving mitochondrial integrity, maintaining mitochondria-ER contacts, and supporting autophagic flux. FUNDC1 likewise regulates ER-mitochondria calcium transfer, which is vital for mitochondrial fission, proper mitochondrial function, and cardiac performance. Moreover, loss of FUNDC1 disrupts these processes, leading to impaired mitochondrial dynamics, reduced Ca²⁺ homeostasis, and increased vulnerability to cardiac dysfunction and heart failure [44, 45].

Beyond chemotherapy-induced cardiotoxicity, mitochondrial dysfunction and dysregulated mitophagy also play central roles in ischemia-associated cardiac injury. In this context, myocardial I/R injury promotes cardiomyocyte death through mitochondrial dysfunction and dysregulated mitophagy [46]. During myocardial I/R, both BNIP3L/NIX and BNIP3 contribute to apoptosis and adverse ventricular remodeling, whereas BNIP3 silencing reduces cell death and preserves systolic function in mouse models. Pharmacological interventions, such as diltiazem hydrochloride, can exert cardioprotective effects by downregulating BNIP3L/NIX [47, 48]. In contrast, FUNDC1-mediated mitophagy protects cardiomyocytes during ischemia, and its loss worsens mitochondrial damage [49]. Additionally, oxidation and depletion of cardiolipin during myocardial I/R impair mitochondrial function, leading to increased oxidative stress, cell death, and tissue injury [50].

The evidence shows that other mitophagy receptors similarly contribute to protecting cardiomyocytes. BCL2L13 supports mitochondrial quality control under stress by promoting mitophagy and mitochondrial fission, and its loss worsens cardiac dysfunction. In vivo, the deficiency or mutation of the regulatory Ser272 phosphorylation site impairs pressure overload–induced mitophagy, aggravating cardiac dysfunction following transverse aortic constriction [23]. AMBRA1 encourages cardiomyocyte survival through autophagy via Beclin1 and the AMP-activated protein kinase/mammalian target of rapamycin (AMPK/mTOR) pathway [51]. Meanwhile, PHB2 regulates cardiac fatty acid oxidation, and its deficiency causes lipid buildup, mitochondrial problems, and early-onset heart failure [52]. These findings highlight the intricate network of protective mechanisms that help limit cardiomyocyte damage under stress, pointing to potential therapeutic targets.

Coronary artery disease (CAD) is the leading cause of morbidity and mortality worldwide, characterized by atherosclerotic plaque accumulation in coronary arteries that impairs myocardial blood flow [53]. In CAD, BNIP3 is epigenetically upregulated through promoter hypomethylation and correlates with oxidative stress markers, suggesting a role in stress-induced mitochondrial dysfunction and endothelial injury [54]. Moreover, AMBRA1 has been identified among autophagy-related genes associated with CAD in bioinformatic analyses, although its specific functional role in this context remains unclear [55]. Increased expression of BCL2L13 has been reported in acute myocardial infarction associated with CAD, where it is linked to enhanced oxidative stress and cardiomyocyte apoptosis [56]. In addition, altered expression of specific circular RNAs in peripheral blood mononuclear cells has been associated with mitophagy-related pathways, suggesting a link between RNA-mediated regulation and mitochondrial quality control [57]. Impaired mitophagy has been further implicated in disease progression, as reduced BNIP3L/NIX activity in macrophages promotes oxidized low-density lipoprotein-induced pyroptosis and plaque instability [58]. Importantly, remodeling of cardiolipin has been implicated in the pathogenesis of CAD, where its oxidation and depletion promote mitochondrial dysfunction, inflammation, and apoptosis, while its externalization can facilitate mitophagy and exert protective effects [59].

Atrial fibrillation is the most prevalent cardiac arrhythmia and represents a major risk factor for stroke [60]. Impaired mitophagy has been observed in patients with chronic atrial fibrillation, characterized by defective delivery of mitochondria to autophagosomes, leading to accumulation of dysfunctional mitochondria [61]. Consistently, in an in vivo rat model of atrial tachyarrhythmia, receptor-mediated mitophagy, mediated by BNIP3, BNIP3L/NIX, and FUNDC1, is significantly downregulated, indicating impaired mitochondrial quality control [62]. In contrast, in an in vitro angiotensin II-stimulated atrial myocyte model, activation of FUNDC1-mediated mitophagy via ULK1-dependent phosphorylation restores mitochondrial function and protects against atrial remodeling [63].

Heart failure is a highly prevalent clinical syndrome affecting over 60 million people worldwide, characterized by impaired cardiac function and driven by complex mechanisms including mitochondrial dysfunction and defective quality control [64]. Receptor-mediated mitophagy is markedly impaired in heart failure, as evidenced by the downregulation of key mitochondrial receptors, including BNIP3, BNIP3L/NIX, and FUNDC1 [65]. In particular, reduced FUNDC1 expression is mechanistically linked to disease progression through impaired mitophagy, disrupted mitochondria-ER coupling, altered calcium homeostasis, and consequent mitochondrial dysfunction, ultimately contributing to cardiomyocyte injury [45, 66]. Similarly, BCL2L13-mediated mitophagy exerts cardioprotective effects in pressure overload, as its activation promotes mitochondrial quality control and preserves cardiac function [67]. In parallel, emerging evidence identifies PHB2 as a key regulator of cardiac fatty acid metabolism, whose deficiency impairs mitochondrial bioenergetics and promotes heart failure development [52]. Additionally, alterations in cardiolipin content and acyl chain composition further compromise mitochondrial function by disrupting respiratory chain organization and bioenergetic efficiency [68].

**Fig. 2:**
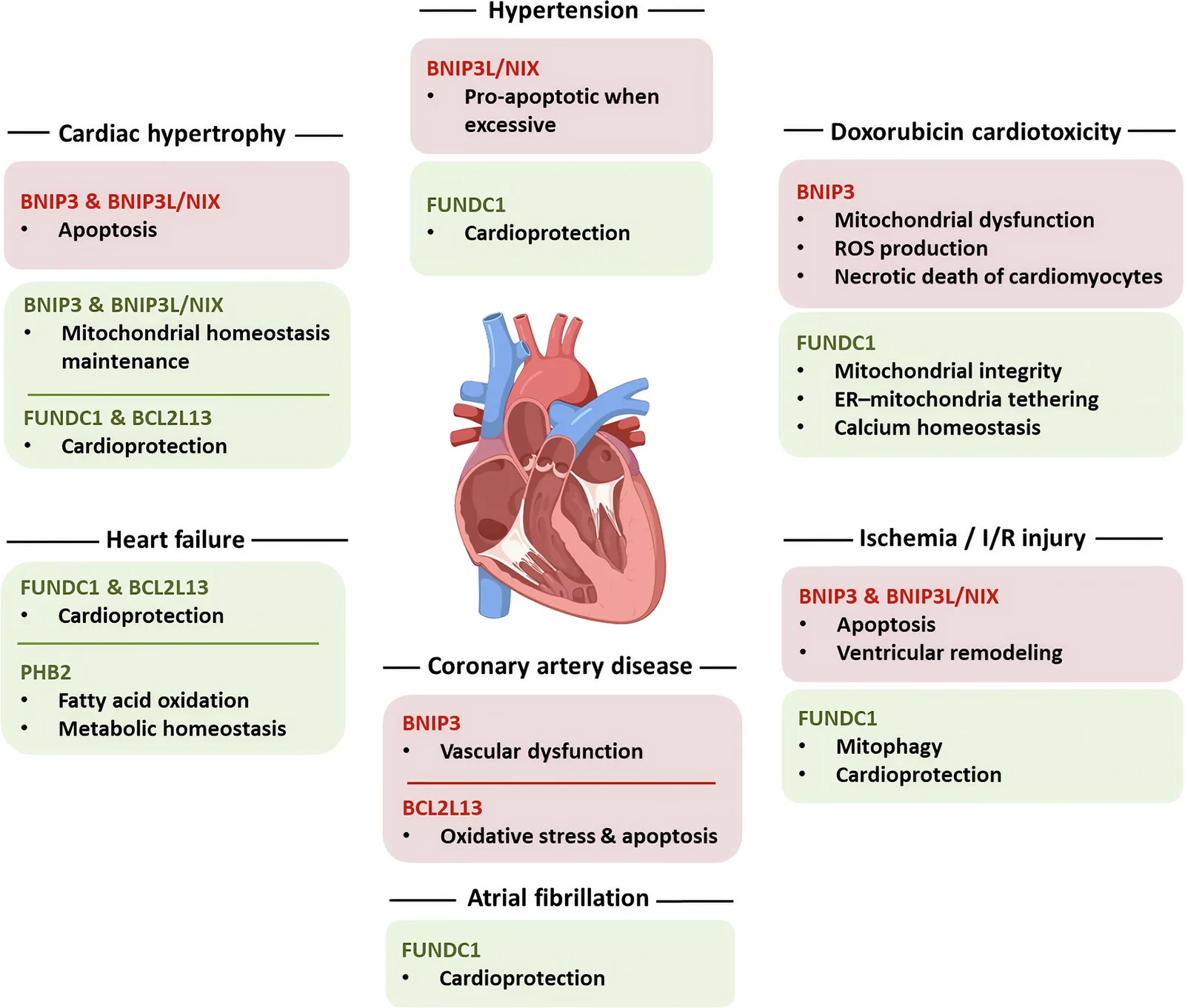
Context-dependent roles of mitophagy receptors in cardiovascular stress and disease. Schematic overview illustrating the differential and often opposing functions of mitophagy receptors in cardiomyocytes under distinct pathological conditions. BNIP3 and BNIP3L/NIX are associated with mitochondrial dysfunction, apoptosis, and adverse remodeling under hypertrophic stress, cardiotoxicity, and ischemic injury, whereas FUNDC1 predominantly supports mitochondrial integrity, mitophagy, and cardioprotection. Other mitophagy receptors, including BCL2L13 and PHB2, may also contribute to mitochondrial quality control under stress conditions. Together, these pathways highlight the context-dependent roles of mitophagy receptors in shaping cardiomyocyte fate and cardiovascular disease progression. Abbreviations: ROS, reactive oxygen species.

As illustrated in Fig. 2, mitophagy receptors exert highly context-dependent and often opposing effects on cardiomyocyte fate, underscoring the need for finely balanced mitochondrial quality control rather than uniform activation or suppression of mitophagy. As mitochondria-rich, terminally differentiated cells with minimal regenerative capacity, cardiomyocytes require a precisely tuned and tightly regulated mitophagy machinery to maintain mitochondrial quality, meet continuous energetic demands, and preserve cellular integrity under stress. Dysregulation of receptor-mediated mitophagy, whether excessive or insufficient, compromises mitochondrial fitness, precipitates irreversible cardiomyocyte loss, and drives maladaptive cardiac remodeling, thereby positioning mitophagy receptors as a particularly compelling target for therapeutic intervention in cardiovascular disease.

## Neurological diseases

Intracerebral hemorrhage (ICH) is a highly lethal form of stroke that induces secondary brain injury driven by hematoma-associated edema, oxidative stress, and inflammation, resulting in neuronal loss and neurological deficits [69]. BNIP3L/NIX is broadly expressed across tissues but is upregulated in neurons within the perihematomal region in a rat model of ICH. In this context, it colocalizes with active caspase-3 and is not detected in astrocytes or oligodendrocytes, suggesting a neuron-enriched pro-apoptotic role. This role likely involves disrupting BCL-2 complexes, increasing mitochondrial permeability, and activating caspase-3, which contributes to ICH-related apoptosis [70]. Although both BNIP3L/NIX and BNIP3 are upregulated after ICH, only BNIP3 expression promotes mitophagy, which reduces apoptosis [71].

In spontaneous subarachnoid hemorrhage, a condition with high rates of disability and mortality, early brain injury is the main cause of death [72]. BNIP3L/NIX is upregulated in neurons in a rat spontaneous subarachnoid hemorrhage model, reaching its peak 24 h after injury. Its overexpression reduces mitochondrial damage, neuronal apoptosis, and cognitive deficits, while knockdown worsens outcomes, indicating a protective role through mitophagy [73]. In cerebral ischemia, where reduced blood flow causes lasting brain damage [74], mitophagy supports neuronal survival [75]. BNIP3L/NIX overexpression can rescue ischemic damage in a Parkin-independent manner, supporting receptor-mediated mitophagy pathways [76]. Similarly, FUNDC1 protects against cerebral ischemia/reperfusion (I/R) injury by AMPK-driven upregulation, thereby enhancing LC3-mediated mitochondrial clearance and suppressing apoptotic signaling [77]. Conversely, BCL2L13 functions as a pro-apoptotic mediator in I/R injury, while genetic or microRNA-mediated suppression alleviates damage [78]. Under ischemic stress, BNIP3 promotes dysfunctional mitophagy and exacerbates neuronal injury in neonatal stroke models. Although BNIP3L/NIX is upregulated in BNIP3-deficient neurons, it does not compensate for BNIP3 loss in mitophagy regulation, indicating BNIP3 drives defective mitophagy under ischemia/hypoxia, while BNIP3L/NIX sustains basal mitophagy under stress [79].

Traumatic brain injury (TBI), which usually results from external mechanical force, typically triggers complex responses in the brain. While the primary injury causes direct tissue damage, the secondary injury largely influences neurological recovery [80]. In a rat TBI model, BNIP3L/NIX expression decreased after injury, promoting autophagy and reducing apoptosis, edema, and neurological deficits, which suggests a neuroprotective role through the regulation of autophagy and apoptosis [81]. Additionally, BNIP3 inhibition by necrostatin-1 protects neurons and oligodendrocytes from TBI and ischemic brain injury. Both in vivo and In vivo studies show that necrostatin-1 prevents BNIP3 from integrating into mitochondria, preserves mitochondrial function, and decreases cell death [82]. Similarly, cardiolipin-mediated mitophagy helps limit neuronal apoptosis and supports recovery after TBI. Suppression of cardiolipin synthesis or translocation to the OMM attenuates mitophagy and worsens neuronal and functional outcomes [83]. More research is needed to understand the context-specific roles of mitophagy regulators and their potential as therapies for these conditions.

Alzheimer’s disease (AD) is the leading cause of neurocognitive decline worldwide, affecting tens of millions of individuals and representing a growing global health burden [84]. Mitochondrial integrity is essential for synaptic function, and accumulating evidence indicates early synaptic mitochondrial dysfunction as a key contributor to AD pathogenesis [85]. A central defect in mitophagy in AD is reduced PINK1 and Parkin levels, resulting in inadequate clearance of damaged mitochondria and their accumulation [86]. The absence of PINK1 accelerates mitochondrial dysfunction and amyloid plaque deposition, impairing learning and memory in mice, while its overexpression enhances mitophagy and reduces synaptic and cognitive impairments [87]. In addition to this well-known pathway, recent evidence emphasizes the significance of alternative mitophagy mechanisms in neurons. Notably, serum BNIP3L/NIX levels are elevated in individuals with AD dementia. Conversely, serum levels of transcription factor EB (TFEB), a major regulator of lysosomal biogenesis, show the opposite trend, supporting the idea that impairment occurs in the final stage of autophagy-lysosomal degradation. BNIP3L/NIX levels also correlate with disease severity and markers of neurodegeneration [88]. Other mitophagy regulators such as AMBRA1, FUNDC1, and BCL2L13 are downregulated in AD, contributing to mitochondrial dysfunction and neuronal loss. Specifically, FUNDC1 reduction is associated with increased neuronal apoptosis and mitochondrial issues, indicating its neuroprotective role, while FKBP8 downregulation worsens mitochondrial dysfunction and tau toxicity in AD [89–91]. Compounds that induce mitophagy, like urolithin A and actinonin, can address these defects by increasing levels of AMBRA1, PINK1, BCL2L13, and other mitophagy-related proteins [92]. In contrast to protective factors, BNIP3, activated by β-amyloid-induced oxidative stress, promotes mitochondrial dysfunction and neuronal death, with its knockdown reducing β-amyloid toxicity [93]. Overall, mitophagy plays a central yet complex role in AD development, with mitophagy receptors exhibiting both protective and harmful effects.

Despite etiological heterogeneity, Parkinson’s disease (PD) converges on common pathogenic pathways, and mitochondrial dysfunction plays a key role in both sporadic and familial PD [94]. Among proteins associated with PD, PINK1 and Parkin have been extensively studied for their role in maintaining mitochondrial health through mitophagy. Mutations that cause loss of function in either gene are the most common causes of autosomal recessive and early-onset PD [95]. Recent research shows that dimethyl fumarate activates the BNIP3/PINK1/Parkin axis to enhance mitophagy and exert neuroprotective effects in PD models [96]. BNIP3 stabilizes full-length PINK1 on the OMM, thereby facilitating Parkin recruitment and providing partial compensation under PINK1-deficient conditions [97]. In models of CCCP-induced mitophagy, BNIP3L/NIX facilitates mitochondrial depolarization and Parkin translocation, and its inhibition, such as by complex I inhibitors, impairs mitophagy and increases vulnerability of dopaminergic neurons [98, 99]. Evidence from *Drosophila* indicates that FUNDC1 can rescue PINK1-deficient phenotypes via a DRP1-dependent, autophagy-independent mechanism, raising the possibility that FUNDC1 similarly modulates mitochondrial dynamics beyond canonical receptor-mediated mitophagy in human cells [100]. In addition to receptor-mediated mitophagy pathways, alterations in mitochondrial lipid homeostasis also contribute to PD pathogenesis. In this context, pathological cardiolipin remodeling mediated by acyl-CoA:lysocardiolipin acyltransferase-1 (ALCAT1) promotes oxidative stress, mitochondrial dysfunction, and impaired mitophagy, whereas its inhibition restores Parkin recruitment and protects dopaminergic neurons from degeneration [101]. These findings underscore the potential of targeting multiple mitophagy receptors to restore mitochondrial health and safeguard dopaminergic neurons in PD.

Across acute brain injury and chronic neurodegenerative disorders, evidence converges on a context-dependent role of mitophagy receptors in regulating neuronal vulnerability rather than uniformly promoting protection. Findings from hemorrhagic, ischemic, traumatic, and neurodegenerative conditions indicate that both insufficient and excessive receptor-mediated mitophagy are detrimental, revealing a narrow homeostatic window for mitochondrial turnover in neurons. Neuronal function critically depends on sustained mitochondrial performance to support synaptic transmission, axonal transport, and calcium signaling within highly polarized cellular architectures. In this setting, receptor-mediated mitophagy operates as a finely tuned quality control system integrating metabolic and injury-related cues with long-term neuronal maintenance. Disruption of this balance propagates mitochondrial dysfunction, network destabilization, and progressive neurological decline, positioning mitophagy receptors as central determinants of disease evolution and attractive, context-sensitive therapeutic targets in neurological disorders.

## Cancer

Cancer is the second leading cause of death in the U.S., with certain types increasing among younger adults. Despite advances in treatments, progress in prevention is slow, and access to care remains unequal [102]. Understanding molecular regulators of tumor survival and death is essential for developing more effective therapies. Mitophagy plays a dual role in cancer, either promoting tumor progression and metastasis or acting tumor-suppressively, depending on the cellular context and specific receptors involved. It supports cancer stem cell maintenance, chemoresistance, metabolic reprogramming, and immune evasion, while in some cases it eliminates damaged mitochondria to reduce ROS and tumor growth [103, 104]. Here, we present a review of the current knowledge on the critical role of mitophagy receptors in regulating cancer cell apoptosis and their response to tumor microenvironmental stresses. Given the extensive and context-dependent nature of mitophagy regulation across cancers, we therefore provide a focused overview of key receptor pathways, while a comprehensive receptor-specific dissection lies beyond the scope of this review.

**Fig. 3:**
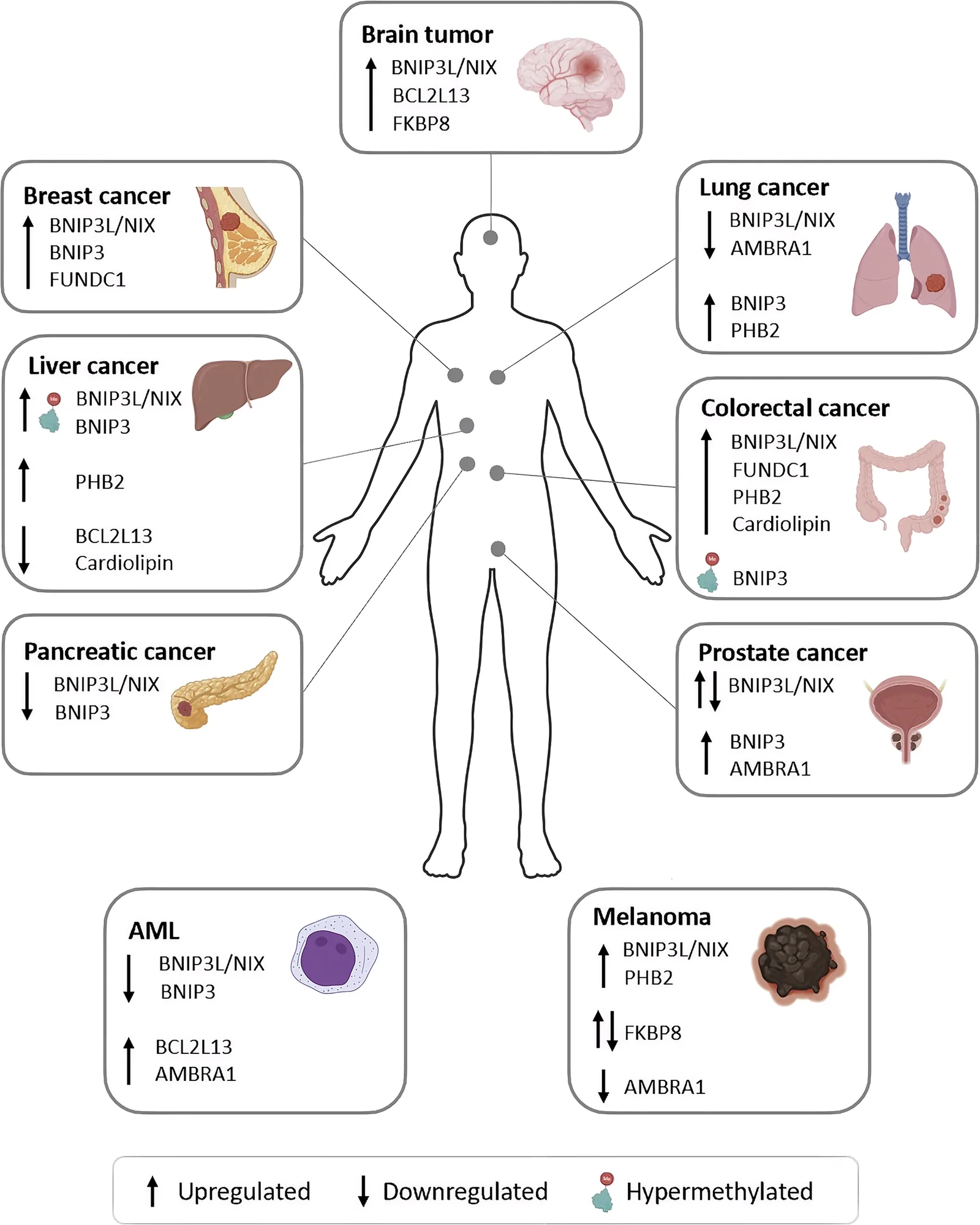
Expression patterns of mitophagy receptors in cancer. Overview of differential regulation of mitophagy receptors across cancer types, highlighting heterogeneous, bidirectional, and conserved expression patterns. AML, acute myeloid leukemia.

Across malignancies, components of the mitophagy machinery display strikingly heterogeneous and often opposing expression patterns, underscoring their strong context dependence in cancer (Fig. 3). In hematological and several solid tumors, BNIP3L/NIX and BNIP3 are frequently downregulated or epigenetically silenced, particularly in acute myeloid leukemia [105, 106], colorectal [107, 108], liver [109] and lung cancer [110], pancreatic ductal adenocarcinoma [111, 112], and prostate cancer [113]. Conversely, both receptors can be induced in hypoxic niches, cancer stem cell populations, or aggressive tumor subtypes, including glioblastoma [114, 115], breast cancer [116, 117], colorectal cancer stem cells [118], liver [119, 120], non-small-cell lung cancer [121], metastatic melanoma [122], and advanced prostate cancer [123, 124]. Beyond BNIP3 family members, other mitophagy regulators show similarly tumor-specific regulation, with BCL2L13, FKBP8, and AMBRA1 being variably upregulated [125–129], suppressed [130–133], or exhibiting bidirectional regulation [132], depending on tumor type, disease stage, and microenvironmental cues. Among mitophagy-associated receptors, FUNDC1 and PHB2 appear to be the most consistently upregulated across diverse tumor types [134–139]. Notably, tumor-associated remodeling of mitochondrial lipid composition is evident, with divergent cardiolipin alterations observed in colorectal versus hepatocellular carcinoma [140, 141]. Together, these observations highlight that mitophagy-related pathways are not uniformly pro- or anti-tumorigenic, but instead are dynamically rewired across cancer types, stages, and cellular states.

A defining feature of mitophagy in cancer is its integration with metabolic rewiring and stress adaptation rather than simple mitochondrial damage clearance. Tumor cells continuously adjust mitochondrial mass, bioenergetic output, and redox balance to survive fluctuating oxygen and nutrient availability. In this context, distinct receptors contribute to reshaping mitochondrial turnover in a stage- and microenvironment-dependent manner, coordinating mitochondrial dynamics, apoptotic priming, and metabolic plasticity. Overall, tumor-suppressive functions are most frequently reported for BNIP3 family members, whereas PHB2 more consistently supports tumor growth, bioenergetic maintenance, and therapy resistance across multiple tumor types, highlighting receptor-specific functional bias despite strong context dependence (Table 1) [105–109, 112, 114–119, 122, 125–128, 131, 134, 136–139, 142–161]. Collectively, these findings highlight mitophagy receptors as central modulators of cancer cell fate and promising candidates for prognostic stratification and therapeutic targeting.

**Table 1:** Functional and clinical relevance of mitophagy receptors across cancer types.

Cancer type	Mitophagy receptor	Functional role/Context	Key cellular effects	Clinical relevance	Ref.
AML	BNIP3L/NIX	Context-dependent	Mitochondrial apoptosis; drug sensitivity	Sensitization to mitochondrial drugs	105, 143
AML	BNIP3	Tumor suppressive	DAC-induced apoptosis	Improved prognosis	106, 144
AML	BCL2L13	Anti-apoptotic	Mitochondrial membrane stabilization	Leukemic cell survival	125
AML	AMBRA1	Context-dependent	Disease stage dynamics	Treatment response	126
AML	PHB2	Pro-survival	Mitophagy enhancement	Poor prognosis	145
Brain tumor	BNIP3L/NIX	Dual (grade-dependent)	Mitophagy; glioblastoma stem cell survival	Grade- and stemness-associated resistance	114, 115
Brain tumor	BNIP3	Context-dependent	Hypoxia-regulated cell death; nuclear sequestration	Apoptosis resistance	146, 147
Brain tumor	BCL2L13	Pro-survival	Enhanced proliferation and invasion	Tumor progression and aggressiveness	127
Brain tumor	FKBP8	Pro-survival	Apoptosis and autophagy inhibition	Tumor cell survival	128
Brain tumor	PHB2	Pro-survival	PARL–PGAM5–PINK1–Parkin mitophagy	Radioresistance	142
Brain tumor	Cardiolipin	Pro-survival	Mitochondrial membrane integrity	Disruption induces apoptotic cell death	148
Breast cancer	BNIP3L/NIX	Tumor suppressive	Apoptosis; anoikis; immune modulation	Favorable prognosis	116
Breast cancer	BNIP3	Stress-associated	Hypoxia-induced stress responses	Aggressive subtypes	117, 149
Breast cancer	FUNDC1	Pro-tumorigenic	Proliferation; migration; invasion	Poor prognosis	134
Breast cancer	AMBRA1	Pro-metastatic	TGFβ-driven EMT; invasion	Metastatic progression	150
Breast cancer	PHB2	Pro-proliferative	Cell cycle progression	Tumor growth	151
Colorectal cancer	BNIP3L/NIX	Pro-survival (CSCs)	Mitophagy enhancement	Chemoresistance	118
Colorectal cancer	BNIP3	Tumor suppressive	Hypoxia-induced apoptosis	Therapy response	107, 108
Colorectal cancer	PHB2	Pro-survival	OXPHOS maintenance	Tumor progression	136
Liver cancer	BNIP3L/NIX	Context-dependent	Mitophagy; metabolic reprogramming	Stemness regulation	119
Liver cancer	BNIP3	Context-/stage-dependent	Apoptosis; anoikis resistance; mitophagy-dependent metabolic control	Stage-dependent prognostic impact	109, 152, 153
Liver cancer	FUNDC1	Stage-dependent	Stress-adaptive mitophagy	Suppression of inflammasome-driven tumorigenesis	154
Liver cancer	PHB2	Pro-survival	Cell cycle progression	Chemoresistance	137
Lung cancer	BNIP3L/NIX	Pro-apoptotic	Stress-induced accumulation	Arsenic-induced cytotoxicity	155
Lung cancer	AMBRA1	Tumor suppressive	Growth inhibition; apoptosis	Better survival	131
Lung cancer	PHB2	Pro-survival	Parkin-mediated mitophagy	Tumor growth	138
Melanoma	BNIP3L/NIX	Tumor suppressive	Autophagic cell death	Improved survival	122
Melanoma	AMBRA1	Tumor suppressive	Invasion and metastasis restraint	Aggressive disease when lost	156
Melanoma	PHB2	Pro-survival	Tumor cell survival	Poor outcome	139
Pancreatic cancer	BNIP3L/NIX	Dual	Mitophagy; invasion	Therapy resistance	157, 158
Pancreatic cancer	BNIP3	Tumor suppressive	Hypoxia-induced cell death	DAC sensitivity	112
Prostate cancer	BNIP3L/NIX	Tumor suppressive	Growth inhibition	Improved prognosis	159
Prostate cancer	BNIP3	Pro-survival	Hypoxia-induced autophagy	Therapy resistance	160
Prostate cancer	PHB2	Pro-metastatic	Cell migration	Aggressive disease	161

*AML* acute myeloid leukemia, *CSCs* cancer stem cells, *DAC* decitabine, *EMT* epithelial-to-mesenchymal transition, *OXPHOS* oxidative phosphorylation, *PARL* presenilin-associated rhomboid-like protein, *PGAM5* phosphoglycerate mutase family member 5, *PINK1* PTEN-induced kinase 1, *TGFβ* transforming growth factor beta.

## Metabolic diseases

Metabolic syndrome, also known as syndrome X or insulin resistance, includes abdominal obesity, insulin resistance, hypertension, and dyslipidemia, increasing the risk of cardiovascular disease [162]. At the cellular level, metabolic disorders are characterized by chronic mitochondrial stress rather than acute organelle damage, placing mitochondrial quality control at the center of metabolic flexibility and endocrine regulation. In this context, mitophagy emerges not only as a protective mechanism but as a finely tuned process whose dysregulation contributes to insulin resistance, impaired glucose handling, and tissue-specific metabolic dysfunction [163].

In pancreatic β-cells, mitophagy is increasingly recognized as a central adaptive mechanism that preserves mitochondrial bioenergetics required for glucose-stimulated insulin secretion and glycemic control. Both ubiquitin- and receptor-mediated pathways are engaged in response to metabolic, inflammatory, and oxidative stress, highlighting the integrated nature of β-cell mitochondrial quality control [164]. Consistent with this protective role, impairment of the mitophagy regulator C-type lectin domain family 16, member A (CLEC16A) exacerbates inflammatory mitochondrial damage, β-cell apoptosis, and hyperglycemia, whereas its restoration confers protection against inflammatory injury [165]. Mitophagy receptors exert distinct and context-dependent effects on cell survival and insulin secretion. BNIP3L/NIX mediates β-cell death under pancreatic duodenal homeobox (PDX1) deficiency, whereas its ablation protects β-cells, enhances insulin secretion, and prevents hyperglycemia [166]. In contrast, PHB2 is essential for proper mitochondrial function and β-cell survival, as its deficiency results in β-cell failure and disruptions in glucose metabolism [167]. These findings illustrate that balanced mitochondrial turnover, rather than global mitophagy activation, is critical for β-cell resilience.

Skeletal muscle further exemplifies the tissue-specific complexity of mitophagy regulation in metabolic disease. In myotubes, BNIP3L/NIX expression triggers endoplasmic/sarcoplasmic reticulum calcium release, mitochondrial fragmentation, and mitophagy, reducing insulin sensitivity, effects reversed by knockdown or protein kinase A (PKA)-mediated Ser212 phosphorylation [168]. Both BNIP3L/NIX and FUNDC1 are activated under lipotoxic stress, such as palmitate exposure or high-fat diet, with BNIP3L/NIX exacerbating insulin resistance and FUNDC1 conferring metabolic protection [168, 169]. Beyond skeletal muscle, whole-body or adipose tissue–specific ablation of FUNDC1 impairs mitophagy in white adipose tissue, leading to mitochondrial dysfunction, increased ROS production, adipose tissue inflammation, and aggravated high-fat diet–induced obesity and insulin resistance [170]. Additionally, BCL2L13 plays a key role in maintaining skeletal muscle function by regulating ER-mitochondria calcium signaling, where its loss leads to impaired muscle structure, reduced exercise capacity, mitochondrial dysfunction, and disturbed calcium homeostasis [171].

In addition to tissue-specific effects, certain mitophagy receptors also influence systemic metabolic regulation. In this context, AMBRA1 deficiency has been linked to a prediabetic metabolic profile in mice, including hyperglycemia, insulin resistance, and impaired fatty acid oxidation, highlighting its contribution to whole-body metabolic balance [172]. Chronic nutrient excess and oxidative stress impair mitochondrial bioenergetics by promoting cardiolipin remodeling and peroxidation, resulting in reduced respiratory chain activity and ATP production. Such lipid-driven mitochondrial dysfunction further increases the reliance on efficient mitophagy to preserve metabolic flexibility [173]. Consistent with this, BNIP3 drives mitochondrial metabolic remodeling by promoting fatty acid oxidation and elevating histone acetylation, which contributes to cellular senescence [174].

Overall, metabolic diseases reveal mitophagy as a regulator of metabolic adaptability rather than a simple stress-response or quality-control pathway. Through tissue-specific and context-dependent actions, mitophagy receptors integrate mitochondrial turnover with calcium signaling, lipid handling, and insulin responsiveness. Disruption of this finely balanced network compromises metabolic flexibility, predisposing tissues to insulin resistance, β-cell dysfunction, and impaired muscle performance, and positioning mitophagy at the core of metabolic disease pathophysiology.

## Hematological disorders

Erythropoiesis represents one of the best-characterized examples of developmentally programmed mitophagy, where mitochondrial clearance is an obligate step of terminal differentiation. This process begins in embryonic life and continues into adulthood, requiring hematopoietic stem cells to generate enucleated erythrocytes [175]. BNIP3L/NIX plays a crucial role in erythropoiesis, as BNIP3L/NIX^−/−^ mice exhibit splenomegaly, erythroblastosis, abnormal erythrocyte morphology, reticulocytosis, and decreased apoptosis during erythroid differentiation, along with blocked mitophagy despite normal autophagy [176, 177]. Failure of this developmental mitophagy program has direct clinical consequences, linking mitochondrial retention to ineffective erythropoiesis and anemia. Clinically, BNIP3L/NIX expression is decreased in high-risk myelodysplastic syndrome, correlating with the severity of anemia and the presence of ring sideroblasts, linking defective mitophagy to anemia development [178]. The mitophagy receptor PHB2 supports mitochondrial function in erythroid progenitors, and its depletion disrupts mitochondrial biogenesis, protein synthesis, and differentiation [179].

Beyond stem cell disorders, disrupted receptor-mediated mitophagy also contributes to inherited anemias characterized by proteotoxic and oxidative stress. In β-thalassemia, caused by mutations in the β-globin gene, reduced or absent β-globin chain synthesis leads to ineffective erythropoiesis and anemia [180]. In this context, AMBRA1 promotes autophagic clearance of excess α-globin, and its dysfunction due to specific missense mutations contributes to oxidative stress, impaired erythroid maturation, and more severe disease phenotypes [181]. Conversely, BNIP3 drives mitochondrial fragmentation and excessive autophagy, contributing to apoptosis and oxidative damage in β-thalassemia erythroid progenitors [182].

FUNDC1 protects against renal anemia by preserving mitochondrial quality and suppressing inflammation in renal erythropoietin-producing cells under stress. Its deficiency impairs mitophagy, increases ROS and cytokines, promotes myofibroblast transformation, reduces erythropoietin production, and worsens renal fibrosis [183].

Hematological disorders underscore receptor-mediated mitophagy as a core determinant of erythroid maturation and hematopoietic integrity. Unlike cancer or degenerative diseases, defective mitophagy in the hematopoietic system primarily disrupts developmental programs rather than inducing irreversible cell loss, resulting in ineffective hematopoiesis, mitochondrial dysfunction, and anemia. These findings position mitophagy receptors as critical developmental regulators whose precise temporal and lineage-specific control is essential for maintaining hematological homeostasis.

## Inflammatory diseases and autoimmune disorders

Inflammatory bowel diseases, notably Crohn’s disease and ulcerative colitis, are chronic inflammatory disorders of the digestive tract caused by a complex interplay of genetic, environmental, and immune factors [184]. Mitochondrial quality control, especially mitophagy, plays a key role in inflammatory bowel disease development. BNIP3L/NIX expression is increased in inflamed intestinal tissues, and its loss worsens colitis by promoting mitochondrial dysfunction and inflammation [185]. Similarly, BNIP3-mediated mitophagy maintains intestinal epithelial cell homeostasis, and its dysregulation increases inflammation and cell death in ulcerative colitis [186]. In contrast, AMBRA1 is upregulated in inflamed tissues of both inflammatory bowel disease patients and colitis mouse models, where its removal decreases cytokine production and enhances response to infliximab [187]. In Crohn’s disease, increased FKBP8-myosin light chain kinase 1 (MLCK1) interaction, observed in patient biopsies, promotes MLCK1 recruitment to intercellular junctions, contributing to epithelial barrier dysfunction and sustained intestinal inflammation [188].

Beyond the gut, BNIP3L/NIX-mediated mitophagy is crucial for immune cells, especially natural killer (NK) cells that eliminate infected or transformed targets [189]. During viral infection, proliferating NK cells gather dysfunctional mitochondria and ROS, which trigger mitophagy to support survival during their transition to memory cells. While both proteins play non-redundant roles in NK cell memory formation, BNIP3 seems particularly important, as its loss hampers mitophagy and causes mitochondrial damage, highlighting its role in NK cell health and longevity [190]. In addition to immune cells, platelets rely on BNIP3L/NIX-mediated mitophagy to preserve mitochondrial integrity required for proper activation and hemostatic function [191].

Rather than acting solely as a downstream stress response, receptor-mediated mitophagy actively shapes immune cell function, epithelial resilience, and the persistence of inflammatory responses by regulating mitochondrial integrity across multiple cellular compartments. As this field continues to evolve, mitophagy receptors provide a new conceptual framework for understanding how mitochondrial dysregulation contributes to chronic inflammation and immune imbalance.

## Conclusions and future perspective

Given the essential roles of mitochondria in energy metabolism, signaling, and apoptosis, disruptions in receptor-mediated mitophagy contribute to a wide spectrum of human diseases [1]. Rather than acting as a uniform quality-control pathway, mitophagy is governed by specialized receptors that function in a tissue- and condition-dependent manner. Understanding this receptor-specific organization is therefore key to translating mitophagy biology into effective therapeutic strategies.

A paradigmatic example of mitophagy specialization is erythropoiesis, where mitochondrial clearance is a genetically programmed developmental event rather than a damage response. The strict requirement for BNIP3L/NIX in erythroid differentiation illustrates how programmed mitophagy directly shapes cell fate decisions and, when disrupted, contributes to ineffective hematopoiesis [105, 176–178]. These observations suggest that restoring BNIP3L/NIX activity could improve erythroid maturation, while also highlighting the need for precise control, as dysregulated mitophagy may disrupt hematopoietic balance. In cancer, the dual behavior of BNIP3L/NIX, epigenetic silencing in certain contexts and hypoxia-driven induction in others (Table 1), indicates a role in defining mitochondrial stress thresholds rather than acting as a classical tumor suppressor or oncogene.

In contrast to the developmental role of BNIP3L/NIX, its homolog BNIP3 functions as a hypoxia-responsive receptor that primarily amplifies cellular stress under conditions of excessive mitochondrial damage. In acute hypoxic or ischemic injury of the brain and heart, BNIP3-driven mitophagy and apoptosis likely reflect an unchecked stress response, suggesting that carefully timed modulation of BNIP3 activity could limit irreversible mitochondrial loss [47]. Beyond hypoxia, BNIP3 intersects with the PINK1-Parkin axis, further emphasizing its role as a stress-amplifying node in mitochondrial quality control [96, 97]. In cancer, frequent epigenetic silencing of BNIP3 indicates selective pressure to suppress hypoxia-induced cell death, while reactivation restores apoptotic competence and chemosensitivity (Fig. 3 and Table 1). These opposing roles suggest that BNIP3 should be targeted in a time- and tissue-specific manner, aiming to modulate its stress-sensing function rather than uniformly inhibiting or activating the pathway.

Protective mitophagy is most consistently observed in pathways regulated by FUNDC1. In acute ischemic settings, FUNDC1 activity is associated with preserved mitochondrial integrity and reduced tissue injury in both the heart and brain [49, 77]. In cancer, FUNDC1 expression is increased in several tumor types, where it is linked to enhanced stress adaptation and, in certain contexts, therapy resistance, particularly in later disease stages (Fig. 3 and Table 1). These observations suggest that any therapeutic modulation of FUNDC1 should be carefully controlled and context-dependent, favoring short-term, stress-adapted approaches rather than prolonged mitophagy induction.

In addition to stress responses, some receptors primarily control mitochondrial sensitivity to apoptosis. BCL2L13 supports mitochondrial stability and cellular integrity in differentiated tissues; its upregulation in acute myeloid leukemia and glioblastoma is associated with reduced mitochondrial apoptosis and enhanced tumor cell survival (Fig. 3 and Table 1), often alongside other anti-apoptotic regulators such as FKBP8, which suppresses both apoptosis and autophagy in therapy-resistant tumors [125, 127, 128]. This dual role suggests that therapeutic strategies targeting BCL2L13 would need to be highly selective, aiming to lower apoptotic thresholds in cancer cells while preserving its essential functions in normal tissues.

Functionally, FKBP8 exerts context-dependent cytoprotective effects by coordinating mitophagy with anti-apoptotic BCL-2/BCL-XL signaling, thereby linking mitochondrial quality control to immune regulation, tumor cell survival, and protection against mitochondrial dysfunction and tau toxicity in neurodegenerative contexts [91, 128, 188].

A similar principle applies to AMBRA1, which integrates mitophagy with broader autophagic and metabolic networks. While its tumor-suppressive functions are evident in lung cancer and melanoma [131, 156], its role in breast cancer is instead associated with tumor-promoting processes [150]. These divergent outcomes indicate that AMBRA1 function is not uniform across cancers but instead reflects the oncogenic signaling networks and metabolic state of each tumor type rather than its expression level alone.

At the IMM, PHB2 represents a core mitophagy receptor with essential physiological functions. Severe mitochondrial dysfunction following its loss highlights its essential role in the heart and erythroid lineage [52, 179]. The consistent upregulation of PHB2 across tumor types raises the possibility that certain cancers become dependent on PHB2-mediated mitochondrial maintenance (Fig. 3 and Table 1). Therapeutic strategies will therefore need to distinguish pathological dependence from physiological necessity.

Finally, mitochondrial membrane composition itself emerges as a regulatory layer. Changes in cardiolipin distribution or oxidation have been associated with altered mitochondrial function, enhanced apoptotic susceptibility, and stress-induced tissue damage [83, 148]. This highlights cardiolipin remodeling, rather than global depletion, as a more selective therapeutic target.

Despite substantial progress, important conceptual questions remain unresolved. It is still unclear how multiple mitophagy receptors are coordinated within the same cell, whether they act redundantly or sequentially, and how their activity is tuned across different mitochondrial subpopulations and disease stages. Importantly, most current knowledge derives from studies focusing on individual receptors in isolation, which limits our understanding of their potential cooperation, competition, or hierarchical organization within the broader mitophagy network. Addressing this gap will require systematic approaches that investigate multiple receptors simultaneously within the same experimental and disease context. In particular, future studies should move beyond single-receptor analyses and employ integrative models that enable parallel interrogation of multiple mitophagy receptors within defined disease settings. Such approaches will be essential to resolve whether receptors function redundantly, cooperatively, or hierarchically, and to define how their coordinated activity shapes mitochondrial quality control and cell fate. These efforts should be complemented by the integration of single-cell and spatial approaches with functional in vivo models to better define receptor-specific roles in physiological and pathological contexts. Equally important will be the identification of biomarkers that reflect receptor-driven mitophagy activity in patients, enabling more precise disease stratification and therapeutic monitoring. A deeper understanding of these mechanisms will be essential for translating mitophagy research into safe and effective therapeutic strategies.

In conclusion, mitophagy receptors do not constitute a single, linear quality-control pathway but instead form a modular and dynamically regulated network that integrates mitochondrial clearance with development, stress adaptation, apoptosis, and metabolic rewiring. Future therapeutic efforts should therefore focus on receptor-specific mechanisms, temporal control, and tissue selectivity, aiming to correct pathological mitophagy while preserving its indispensable roles in physiological mitochondrial homeostasis.

## References

[1] Annesley SJ, Fisher PR. Mitochondria in health and disease. Cells. 2019;8:680. 31284394 10.3390/cells8070680 PMC6678092

[2] Onishi M, Yamano K, Sato M, Matsuda N, Okamoto K. Molecular mechanisms and physiological functions of mitophagy. EMBO J. 2021;40:e104705. 33438778 10.15252/embj.2020104705 PMC7849173

[3] Ganley IG, Simonsen A. Diversity of mitophagy pathways at a glance. J Cell Sci. 2022;135:jcs259748. 36504076 10.1242/jcs.259748 PMC10656428

[4] Chen G, Ray R, Dubik D, Shi L, Cizeau J, Bleackley RC, et al. The E1B 19K/Bcl-2-binding protein Nip3 is a dimeric mitochondrial protein that activates apoptosis. J Exp Med. 1997;186:1975–1983. 9396766 10.1084/jem.186.12.1975 PMC2199165

[5] Imazu T, Shimizu S, Tagami S, Matsushima M, Nakamura Y, Miki T, et al. Bcl-2/E1B 19 kDa-interacting protein 3-like protein (Bnip3L) interacts with bcl-2/Bcl-xL and induces apoptosis by altering mitochondrial membrane permeability. Oncogene. 1999;18:4523–9. 10467396 10.1038/sj.onc.1202722

[6] Niemi NM, Friedman JR. Coordinating BNIP3/NIX-mediated mitophagy in space and time. Biochem Soc Trans. 2024;52:1969–79. 39377319 10.1042/BST20221364 PMC11555697

[7] Marinković M, Šprung M, Novak I. Dimerization of mitophagy receptor BNIP3L/NIX is essential for recruitment of autophagic machinery. Autophagy. 2021;17:1232–43. 32286918 10.1080/15548627.2020.1755120 PMC8143235

[8] Bunker EN, Le Guerroué F, Wang C, Strub MP, Werner A, Tjandra N, et al. Nix interacts with WIPI2 to induce mitophagy. EMBO J. 2023;42:e113491. 37621214 10.15252/embj.2023113491 PMC10646555

[9] Zhu Y, Massen S, Terenzio M, Lang V, Chen-Lindner S, Eils R, et al. Modulation of serines 17 and 24 in the LC3-interacting region of Bnip3 determines pro-survival mitophagy versus apoptosis. J Biol Chem. 2013;288:1099–113. 23209295 10.1074/jbc.M112.399345 PMC3542995

[10] Rogov VV, Suzuki H, Marinković M, Lang V, Kato R, Kawasaki M, et al. Phosphorylation of the mitochondrial autophagy receptor Nix enhances its interaction with LC3 proteins. Sci Rep. 2017;7:1131. 28442745 10.1038/s41598-017-01258-6 PMC5430633

[11] Adriaenssens E, Schaar S, Cook ASI, Stuke JFM, Sawa-Makarska J, Nguyen TN, et al. Reconstitution of BNIP3/NIX-mitophagy initiation reveals hierarchical flexibility of the autophagy machinery. Nat Cell Biol. 2025;27:1272–87. 40715440 10.1038/s41556-025-01712-y PMC12339401

[12] Yamashita SI, Arai R, Hada H, Padman BS, Lazarou M, Chan DC, et al. The mitophagy receptors BNIP3 and NIX mediate tight attachment and expansion of the isolation membrane to mitochondria. J Cell Biol. 2025;224:e202408166. 40358358 10.1083/jcb.202408166 PMC12071194

[13] Cao Y, Zheng J, Wan H, Sun Y, Fu S, Liu S, et al. A mitochondrial SCF-FBXL4 ubiquitin E3 ligase complex degrades BNIP3 and NIX to restrain mitophagy and prevent mitochondrial disease. EMBO J. 2023;42:e113033. 36896912 10.15252/embj.2022113033 PMC10308365

[14] Elcocks H, Brazel AJ, McCarron KR, Kaulich M, Husnjak K, Mortiboys H, et al. FBXL4 ubiquitin ligase deficiency promotes mitophagy by elevating NIX levels. EMBO J. 2023;42:e112799. 37102372 10.15252/embj.2022112799 PMC10308357

[15] Nguyen-Dien GT, Kozul KL, Cui Y, Townsend B, Kulkarni PG, Ooi SS, et al. FBXL4 suppresses mitophagy by restricting the accumulation of NIX and BNIP3 mitophagy receptors. EMBO J. 2023;42:e112767. 37161784 10.15252/embj.2022112767 PMC10308361

[16] Niemi NM, Serrano LR, Muehlbauer LK, Balnis CE, Wei L, Smith AJ, et al. PPTC7 maintains mitochondrial protein content by suppressing receptor-mediated mitophagy. Nat Commun. 2023;14:6431. 37833277 10.1038/s41467-023-42069-w PMC10575892

[17] Wei L, Gok MO, Svoboda JD, Kozul KL, Forny M, Friedman JR, et al. Dual-localized PPTC7 limits mitophagy through proximal and dynamic interactions with BNIP3 and NIX. Life Sci Alliance. 2024;7:e202402765. 38991726 10.26508/lsa.202402765 PMC11239977

[18] Nguyen-Dien GT, Townsend B, Kulkarni PG, Kozul KL, Ooi SS, Eldershaw DN, et al. PPTC7 antagonizes mitophagy by promoting BNIP3 and NIX degradation via SCFFBXL4. EMBO Rep. 2024;25:3324–47. 38992176 10.1038/s44319-024-00181-y PMC11316107

[19] Longo M, Bishnu A, Risiglione P, Montava-Garriga L, Cuenco J, Sakamoto K, et al. Opposing roles for AMPK in regulating distinct mitophagy pathways. Mol Cell. 2024;84:4350–4367.e9. 39532100 10.1016/j.molcel.2024.10.025

[20] Chen M, Chen Z, Wang Y, Tan Z, Zhu C, Li Y, et al. Mitophagy receptor FUNDC1 regulates mitochondrial dynamics and mitophagy. Autophagy. 2016;12:689–702. 27050458 10.1080/15548627.2016.1151580 PMC4836026

[21] Li K, Xia X, Tong Y. Multiple roles of mitochondrial autophagy receptor FUNDC1 in mitochondrial events and kidney disease. Front Cell Dev Biol. 2024;12:1453365. 39445333 10.3389/fcell.2024.1453365 PMC11496291

[22] Otsu K, Murakawa T, Yamaguchi O. BCL2L13 is a mammalian homolog of the yeast mitophagy receptor Atg32. Autophagy. 2015;11:1932–3. 26506896 10.1080/15548627.2015.1084459 PMC4824574

[23] Murakawa T, Ito J, Rusu MC, Taneike M, Omiya S, Moncayo-Arlandi J, et al. AMPK regulates Bcl2-L-13-mediated mitophagy induction for cardioprotection. Cell Rep. 2024;43:115001. 39580803 10.1016/j.celrep.2024.115001 PMC11672683

[24] Chen Y, Sternberg P, Cai J. Characterization of a Bcl-XL-interacting protein FKBP8 and its splice variant in human RPE cells. Investig Ophthalmol Vis Sci. 2008;49:1721–7. 18385096 10.1167/iovs.07-1121 PMC2715170

[25] Bhujabal Z, Birgisdóttir ÅB, Sjøttem E, Brenne HB, Øvervatn A, Habisov S, et al. FKBP8 recruits LC3A to mediate Parkin-independent mitophagy. EMBO Rep. 2017;18:947–61. 28381481 10.15252/embr.201643147 PMC5452039

[26] Aguilera MO, Colombo MI. FKBP8, a new member of the PIK3C3/VPS34 complex. Autophagy Rep. 2022;1:291–4. 40396046 10.1080/27694127.2022.2100041 PMC11864611

[27] Hawkins K, Watt M, Gillotin S, Hanspal M, Helley M, Richardson J, et al. Disrupting the interaction between AMBRA1 and DLC1 prevents apoptosis while enhancing autophagy and mitophagy. Biol Open. 2024;13:bio060380. 39469809 10.1242/bio.060380 PMC11625884

[28] Sato M, Sato K. Degradation of paternal mitochondria by fertilization-triggered autophagy in C. elegans embryos. Science. 2011;334:1141–4. 21998252 10.1126/science.1210333

[29] Wei Y, Chiang WC, Sumpter R Jr, Mishra P, Levine B. Prohibitin 2 is an inner mitochondrial membrane mitophagy receptor. Cell. 2017;168:224–238.e10. 28017329 10.1016/j.cell.2016.11.042 PMC5235968

[30] Antón Z, Landajuela A, Hervás JH, Montes LR, Hernández-Tiedra S, Velasco G, et al. Human Atg8-cardiolipin interactions in mitophagy: specific properties of LC3B, GABARAPL2 and GABARAP. Autophagy. 2016;12:2386–403. 27764541 10.1080/15548627.2016.1240856 PMC5172498

[31] Wang L, Qi H, Tang Y, Shen HM. Post-translational modifications of key machinery in the control of mitophagy. Trends Biochem Sci. 2020;45:58–75. 31606339 10.1016/j.tibs.2019.08.002

[32] Grossman W, Jones D, McLaurin LP. Wall stress and patterns of hypertrophy in the human left ventricle. J Clin Invest. 1975;56:56–64. 124746 10.1172/JCI108079 PMC436555

[33] Narula J, Haider N, Virmani R, DiSalvo TG, Kolodgie FD, Hajjar RJ, et al. Apoptosis in myocytes in end-stage heart failure. N Engl J Med. 1996;335:1182–9. 8815940 10.1056/NEJM199610173351603

[34] Yussman MG, Toyokawa T, Odley A, Lynch RA, Wu G, Colbert MC, et al. Mitochondrial death protein Nix is induced in cardiac hypertrophy and triggers apoptotic cardiomyopathy. Nat Med. 2002;8:725–30. 12053174 10.1038/nm719

[35] Chaanine AH, Jeong D, Liang L, Chemaly ER, Fish K, Gordon RE, et al. JNK modulates FOXO3a for the expression of the mitochondrial death and mitophagy marker BNIP3 in pathological hypertrophy and in heart failure. Cell Death Dis. 2012;3:265. 22297293 10.1038/cddis.2012.5 PMC3288347

[36] Diwan A, Wansapura J, Syed FM, Matkovich SJ, Lorenz JN, Dorn GW. 2nd. Nix-mediated apoptosis links myocardial fibrosis, cardiac remodeling, and hypertrophy decompensation. Circulation. 2008;117:396–404. 18178777 10.1161/CIRCULATIONAHA.107.727073 PMC2538800

[37] Gálvez AS, Brunskill EW, Marreez Y, Benner BJ, Regula KM, Kirschenbaum LA, et al. Distinct pathways regulate proapoptotic Nix and BNip3 in cardiac stress. J Biol Chem. 2006;281:1442–8. 16291751 10.1074/jbc.M509056200

[38] Syed F, Odley A, Hahn HS, Brunskill EW, Lynch RA, Marreez Y, et al. Physiological growth synergizes with pathological genes in experimental cardiomyopathy. Circ Res. 2004;95:1200–6. 15539635 10.1161/01.RES.0000150366.08972.7f

[39] Dorn GW. 2nd. Mitochondrial pruning by Nix and BNip3: an essential function for cardiac-expressed death factors. J Cardiovasc Transl Res. 2010;3:374–83. 20559783 10.1007/s12265-010-9174-x PMC2900478

[40] Li W, Yin L, Sun X, Wu J, Dong Z, Hu K, et al. Alpha-lipoic acid protects against pressure overload-induced heart failure via ALDH2-dependent Nrf1-FUNDC1 signaling. Cell Death Dis. 2020;11:599. 32732978 10.1038/s41419-020-02805-2 PMC7393127

[41] Li S, Li X. Mitophagy in hypertensive cardiac hypertrophy: mechanisms and therapeutic implications. J Clin Hypertens. 2025;27:e70127. 10.1111/jch.70127 PMC12358939 40823764

[42] Zhao X, Cui L, Xiao Y, Mao Q, Aishanjiang M, Kong W, et al. Hypertension-associated mitochondrial DNA 4401A>G mutation caused the aberrant processing of tRNAMet, all 8 tRNAs and ND6 mRNA in the light-strand transcript. Nucleic Acids Res. 2019;47:10340–56. 31504769 10.1093/nar/gkz742 PMC6821173

[43] Dhingra R, Margulets V, Chowdhury SR, Thliveris J, Jassal D, Fernyhough P, et al. Bnip3 mediates doxorubicin-induced cardiac myocyte necrosis and mortality through changes in mitochondrial signaling. Proc Natl Acad Sci USA. 2014;111:E5537–E5544. 25489073 10.1073/pnas.1414665111 PMC4280597

[44] He W, Sun Z, Tong G, Zeng L, He W, Chen X, et al. FUNDC1 alleviates doxorubicin-induced cardiotoxicity by restoring mitochondrial-endoplasmic reticulum contacts and blocked autophagic flux. Theranostics. 2024;14:3719–38. 38948070 10.7150/thno.92771 PMC11209712

[45] Wu S, Lu Q, Wang Q, Ding Y, Ma Z, Mao X, et al. Binding of FUN14 domain-containing 1 with inositol 1,4,5-trisphosphate receptor in mitochondria-associated endoplasmic reticulum membranes maintains mitochondrial dynamics and function in hearts in vivo. Circulation. 2017;136:2248–66. 28942427 10.1161/CIRCULATIONAHA.117.030235 PMC5716911

[46] Kulek AR, Anzell A, Wider JM, Sanderson TH, Przyklenk K. Mitochondrial quality control: role in cardiac models of lethal ischemia-reperfusion injury. Cells. 2020;9:214. 31952189 10.3390/cells9010214 PMC7016592

[47] Zhou X, Lu Q, Wang Q, Chu W, Huang J, Yu J, et al. Diltiazem hydrochloride protects against myocardial ischemia/reperfusion injury in a BNIP3L/NIX-mediated mitophagy manner. J Inflamm Res. 2024;17:8905–19. 39575347 10.2147/JIR.S493037 PMC11579144

[48] Diwan A, Krenz M, Syed FM, Wansapura J, Ren X, Koesters AG, et al. Inhibition of ischemic cardiomyocyte apoptosis through targeted ablation of Bnip3 restrains postinfarction remodeling in mice. J Clin Invest. 2007;117:2825–33. 17909626 10.1172/JCI32490 PMC1994631

[49] Xu C, Cao Y, Liu R, Liu L, Zhang W, Fang X, et al. Mitophagy-regulated mitochondrial health strongly protects the heart against cardiac dysfunction after acute myocardial infarction. J Cell Mol Med. 2022;26:1315–26. 35040256 10.1111/jcmm.17190 PMC8831983

[50] Xing Y, Xie SY, Deng W, Tang QZ. Cardiolipin in myocardial ischaemia-reperfusion injury: from molecular mechanisms to clinical strategies. Biomed Pharmacother. 2024;176:116936. 38878685 10.1016/j.biopha.2024.116936

[51] Shi C, Wu J, Fu M, Zhang B, Wang J, Yang X, et al. Ambra1 modulates starvation-induced autophagy through AMPK signaling pathway in cardiomyocytes. Biochem Biophys Res Commun. 2014;452:308–14. 25117440 10.1016/j.bbrc.2014.08.017

[52] Wu D, Jian C, Peng Q, Hou T, Wu K, Shang B, et al. Prohibitin 2 deficiency impairs cardiac fatty acid oxidation and causes heart failure. Cell Death Dis. 2020;11:181. 32165613 10.1038/s41419-020-2374-7 PMC7067801

[53] Shahjehan RD, Sharma S, Dababneh E, Bhutta BS. Coronary artery disease. StatPearls Publishing, 2024. https://www.ncbi.nlm.nih.gov/books/NBK564304/33231974

[54] Lakshmi SVV, Naushad SM, Reddy CA, Saumya K, Rao DS, Kotamraju S, et al. Oxidative stress in coronary artery disease: epigenetic perspective. Mol Cell Biochem. 2013;374:203–11. 23160801 10.1007/s11010-012-1520-7

[55] Lv J, Wang D, Li T. Autophagy-mediated expression clusters are involved in immunity regulation of coronary artery disease. BMC Genom Data. 2022;23:24. 35365066 10.1186/s12863-022-01023-3 PMC8976398

[56] Ding H, Chen W, Chen X. Serum miR-96-5p is a novel and non-invasive marker of acute myocardial infarction associated with coronary artery disease. Bioengineered. 2022;13:3930–43. 35109756 10.1080/21655979.2022.2031392 PMC8973839

[57] Zhou H, Gan X, He S, Wang Y, Zhang S, Chen J, et al. Identification of circular RNA BTBD7_hsa_circ_0000563 as a novel biomarker for coronary artery disease and the functional discovery of BTBD7_hsa_circ_0000563 based on peripheral blood mononuclear cells: a case-control study. Clin Proteom. 2022;19:37. 10.1186/s12014-022-09374-w PMC9630807 36329387

[58] Peng X, Chen H, Li Y, Huang D, Huang B, Sun D. Effects of NIX-mediated mitophagy on ox-LDL-induced macrophage pyroptosis in atherosclerosis. Cell Biol Int. 2020;44:1481–90. 32181963 10.1002/cbin.11343

[59] Wei J, Zhang M, Wang X, Yang K, Xiao Q, Zhu X, et al. Role of cardiolipin in regulating and treating atherosclerotic cardiovascular diseases. Eur J Pharm. 2024;979:176853. 10.1016/j.ejphar.2024.176853 39067567

[60] Nesheiwat Z, Goyal A, Jagtap M. Atrial fibrillation. StatPearls Publishing, 2026. https://www.ncbi.nlm.nih.gov/books/NBK526072/. 30252328

[61] Zhou S, Dai W, Zhong G, Jiang Z. Impaired mitophagy: a new potential mechanism of human chronic atrial fibrillation. Cardiol Res Pr. 2020;2020:6757350. 10.1155/2020/6757350 PMC7547333 33062320

[62] Wu X, Yan X, Ma R, Wu Q, Pan X, Wu Q, et al. Ion channels and atrial fibrillation: mitophagy as a key mediator. Front Physiol. 2025;16:1687578. 41357167 10.3389/fphys.2025.1687578 PMC12675238

[63] Zhu Y, Gu Z, Shi J, Chen C, Xu H, Lu Q. Vaspin attenuates atrial abnormalities by promoting ULK1/FUNDC1-mediated mitophagy. Oxid Med Cell Longev. 2022;2022:3187463. 36425056 10.1155/2022/3187463 PMC9681551

[64] Shams P, Malik A, Chhabra L. Heart Failure (Congestive Heart Failure). StatPearls Publishing, 2026. https://www.ncbi.nlm.nih.gov/books/NBK430873/. 28613623

[65] Svagusa T, Sikiric S, Milavic M, Sepac A, Seiwerth S, Milicic D, et al. Heart failure in patients is associated with downregulation of mitochondrial quality control genes. Eur J Clin Investig. 2023;53:e14054. 37403271 10.1111/eci.14054

[66] Bai X, Zhang Z, Li X, Yang Y, Ding S. FUNDC1: an emerging mitochondrial and MAMs protein for mitochondrial quality control in heart diseases. Int J Mol Sci. 2023;24:9151. 37298100 10.3390/ijms24119151 PMC10252584

[67] Murakawa T, Otsu K. Phosphorylation of BCL2L13 by PRKAA2/AMPKα2 activates mitophagy in pressure-overloaded heart. Autophagy. 2025;21:1382–3. 39995141 10.1080/15548627.2025.2465408 PMC12087641

[68] Mejia EM, Cole LK, Hatch GM. Cardiolipin metabolism and the role it plays in heart failure and mitochondrial supercomplex formation. Cardiovasc Hematol Disord Drug Targets. 2014;14:98–106. 24801725 10.2174/1871529x14666140505123753

[69] Aronowski J, Zhao X. Molecular pathophysiology of cerebral hemorrhage: secondary brain injury. Stroke. 2011;42:1781–6. 21527759 10.1161/STROKEAHA.110.596718 PMC3123894

[70] Rui Y, Ke K, Li L, Zheng H, Xu W, Tan X, et al. Up-regulated expression of Bnip3L after intracerebral hemorrhage in adult rats. J Mol Histol. 2013;44:497–505. 23771482 10.1007/s10735-013-9506-7

[71] Guan R, Li Z, Dai X, Zou W, Yu X, Liu H, et al. Electroacupuncture at GV20‑GB7 regulates mitophagy to protect against neurological deficits following intracerebral hemorrhage via inhibition of apoptosis. Mol Med Rep. 2021;24:492. 33955500 10.3892/mmr.2021.12131 PMC8127033

[72] Ji C, Chen G. Signaling pathway in early brain injury after subarachnoid hemorrhage: news update. In: Applegate RL, Chen G, Feng H, Zhang JH (eds). Brain Edema XVI. Springer International Publishing; Cham: 2016. pp 123-6. 10.1007/978-3-319-18497-5_21 26463934

[73] Zhang J, Yuan G, Liang T, Pan P, Li X, Li H, et al. Nix plays a neuroprotective role in early brain injury after experimental subarachnoid hemorrhage in rats. Front Neurosci. 2020;14:245. 32265644 10.3389/fnins.2020.00245 PMC7108665

[74] Raichle ME. The pathophysiology of brain ischemia. Ann Neurol. 1983;13:2–10. 6299175 10.1002/ana.410130103

[75] Yuan Y, Zhang X, Zheng Y, Chen Z. Regulation of mitophagy in ischemic brain injury. Neurosci Bull. 2015;31:395–406. 26219224 10.1007/s12264-015-1544-6 PMC5563715

[76] Yuan Y, Zheng Y, Zhang X, Chen Y, Wu X, Wu J, et al. BNIP3L/NIX-mediated mitophagy protects against ischemic brain injury independent of PARK2. Autophagy. 2017;13:1754–66. 28820284 10.1080/15548627.2017.1357792 PMC5640199

[77] Cai Y, Yang E, Yao X, Zhang X, Wang Q, Wang Y, et al. FUNDC1-dependent mitophagy induced by tPA protects neurons against cerebral ischemia-reperfusion injury. Redox Biol. 2021;38:101792. 33212415 10.1016/j.redox.2020.101792 PMC7679257

[78] Liu X, Wang X, Zhang L, Zhou Y, Yang L, Yang M. By targeting apoptosis facilitator BCL2L13, microRNA miR-484 alleviates cerebral ischemia/reperfusion injury-induced neuronal apoptosis in mice. Bioengineered. 2021;12:948–59. 33724167 10.1080/21655979.2021.1898134 PMC8806345

[79] Shi RY, Zhu SH, Li V, Gibson SB, Xu XS, Kong JM. BNIP3 interacting with LC3 triggers excessive mitophagy in delayed neuronal death in stroke. CNS Neurosci Ther. 2014;20:1045–55. 25230377 10.1111/cns.12325 PMC6492992

[80] Hawryluk GWJ, Bullock MR. Past, present, and future of traumatic brain injury research. Neurosurg Clin N Am. 2016;27:375–96. 27637391 10.1016/j.nec.2016.05.002

[81] Ma J, Ni H, Rui Q, Liu H, Jiang F, Gao R, et al. Potential roles of NIX/BNIP3L pathway in rat traumatic brain injury. Cell Transpl. 2019;28:585–95. 10.1177/0963689719840353 PMC7103607 30961359

[82] Mu J, Weng J, Yang C, Guan T, Deng L, Li M, et al. Necrostatin-1 prevents the proapoptotic protein Bcl-2/adenovirus E1B 19-kDa interacting protein 3 from integration into mitochondria. J Neurochem. 2021;156:929–42. 32112403 10.1111/jnc.14993

[83] Chao H, Lin C, Zuo Q, Liu Y, Xiao M, Xu X, et al. Cardiolipin-dependent mitophagy guides outcome after traumatic brain injury. J Neurosci. 2019;39:1930–43. 30626699 10.1523/JNEUROSCI.3415-17.2018 PMC6407296

[84] Alzheimer’s Disease International. World Alzheimer Report 2019: Attitudes to Dementia. 2019. https://www.alzint.org/resource/world-alzheimer-report-2019/.

[85] Sheng ZH, Cai Q. Mitochondrial transport in neurons: impact on synaptic homeostasis and neurodegeneration. Nat Rev Neurosci. 2012;13:77–93. 22218207 10.1038/nrn3156 PMC4962561

[86] Oliver DMA, Reddy PH. Small molecules as therapeutic drugs for Alzheimer’s disease. Mol Cell Neurosci. 2019;96:47–62. 30877034 10.1016/j.mcn.2019.03.001 PMC6510253

[87] Du F, Yu Q, Yan S, Hu G, Lue LF, Walker DG, et al. PINK1 signalling rescues amyloid pathology and mitochondrial dysfunction in Alzheimer’s disease. Brain. 2017;140:3233–51. 29077793 10.1093/brain/awx258 PMC5841141

[88] Veverová K, Laczó J, Katonová A, Horáková H, Matušková V, Angelucci F, et al. Alterations of human CSF and serum-based mitophagy biomarkers in the continuum of Alzheimer's disease. Autophagy. 2024;20:1868–78. 38695174 10.1080/15548627.2024.2340408 PMC11262225

[89] Di Rienzo M, Romagnoli A, Refolo G, Vescovo T, Ciccosanti F, Zuchegna C, et al. Role of AMBRA1 in mitophagy regulation: emerging evidence in aging-related diseases. Autophagy. 2024;20:2602–15. 39113560 10.1080/15548627.2024.2389474 PMC11587829

[90] Ma W, Su Y, Zhang P, Wan G, Cheng X, Lu C, et al. Identification of mitochondrial-related genes as potential biomarkers for the subtyping and prediction of Alzheimer’s disease. Front Mol Neurosci. 2023;16:1205541. 37470054 10.3389/fnmol.2023.1205541 PMC10352499

[91] Isei MO, Crockett M, Chen E, Rodwell-Bullock J, Caroll T, Girardi PA, et al. Tau phosphorylation suppresses oxidative stress-induced mitophagy via FKBP8 receptor modulation. PLoS One. 2025;20:e0307358. 39752365 10.1371/journal.pone.0307358 PMC11698316

[92] Schoenherr C, Byron A, Griffith B, Loftus A, Wills JC, Munro AF, et al. The autophagy protein Ambra1 regulates gene expression by supporting novel transcriptional complexes. J Biol Chem. 2020;295:12045–57. 32616651 10.1074/jbc.RA120.012565 PMC7443501

[93] Zhang S, Zhang Z, Sandhu G, Ma X, Yang X, Geiger JD, et al. Evidence of oxidative stress-induced BNIP3 expression in amyloid beta neurotoxicity. Brain Res. 2007;1138:221–30. 17274962 10.1016/j.brainres.2006.12.086

[94] Dawson TM, Dawson VL. Molecular pathways of neurodegeneration in Parkinson’s disease. Science. 2003;302:819–22. 14593166 10.1126/science.1087753

[95] Quinn PMJ, Moreira PI, Ambrósio AF, Alves CH. PINK1/PARKIN signalling in neurodegeneration and neuroinflammation. Acta Neuropathol Commun. 2020;8:189. 33168089 10.1186/s40478-020-01062-w PMC7654589

[96] Pinjala P, Tryphena KP, Kulkarni A, Goswami PG, Khatri DK. Dimethyl fumarate exerts a neuroprotective effect by enhancing mitophagy via the NRF2/BNIP3/PINK1 Axis in the MPP+ iodide-induced Parkinson’s disease mice model. J Alzheimers Dis Rep. 2024;8:329–44. 38405353 10.3233/ADR-230128 PMC10894611

[97] Zhang T, Xue L, Li L, Tang C, Wan Z, Wang R, et al. BNIP3 protein suppresses PINK1 kinase proteolytic cleavage to promote mitophagy. J Biol Chem. 2016;291:21616–29. 27528605 10.1074/jbc.M116.733410 PMC5076832

[98] Ding WX, Ni HM, Li M, Liao Y, Chen X, Stolz DB, et al. Nix is critical to two distinct phases of mitophagy, reactive oxygen species-mediated autophagy induction and Parkin-ubiquitin-p62-mediated mitochondrial priming. J Biol Chem. 2010;285:27879–90. 20573959 10.1074/jbc.M110.119537 PMC2934655

[99] Gao F, Chen D, Si J, Hu Q, Qin Z, Fang M, et al. The mitochondrial protein BNIP3L is the substrate of PARK2 and mediates mitophagy in PINK1/PARK2 pathway. Hum Mol Genet. 2015;24:2528–38. 25612572 10.1093/hmg/ddv017

[100] Xu J, Deng Z, Shang S, Wang C, Han H. FUNDC1 collaborates with PINK1 in regulating mitochondrial Fission and compensating for PINK1 deficiency. Biochem Biophys Res Commun. 2023;687:149210. 37931419 10.1016/j.bbrc.2023.149210

[101] Song C, Zhang J, Qi S, Liu Z, Zhang X, Zheng Y, et al. Cardiolipin remodeling by ALCAT1 links mitochondrial dysfunction to Parkinson’s diseases. Aging Cell. 2019;18:e12941. 30838774 10.1111/acel.12941 PMC6516155

[102] Siegel RL, Giaquinto AN, Jemal A. Cancer statistics, 2024. CA Cancer J Clin. 2024;74:12–49. 38230766 10.3322/caac.21820

[103] Song C, Pan S, Zhang J, Li N, Geng Q. Mitophagy: a novel perspective for insight into cancer and cancer treatment. Cell Prolif. 2022;55:e13327. 36200262 10.1111/cpr.13327 PMC9715364

[104] Dong Y, Zhang X. Targeting cellular mitophagy as a strategy for human cancers. Front Cell Dev Biol. 2024;12:1431968. 39035027 10.3389/fcell.2024.1431968 PMC11257920

[105] Lazarini M, Machado-Neto JA, Duarte AD, Pericole FV, Vieira KP, Niemann FS, et al. BNIP3L in myelodysplastic syndromes and acute myeloid leukemia: impact on disease outcome and cellular response to decitabine. Haematologica. 2016;101:e445–e448. 27443286 10.3324/haematol.2016.142521 PMC5394857

[106] Lazarini M, Machado-Neto J, Duarte ASS, Pericole FV, Favaro P, Barcellos KSA, et al. BNIP3 is downregulated in myelodysplastic syndromes and acute myeloid leukemia and is a potential molecular target for decitabine. Blood. 2012;120:1708.

[107] He J, Pei L, Jiang H, Yang W, Chen J, Liang H. Chemoresistance of colorectal cancer to 5-fluorouracil is associated with silencing of the BNIP3 gene through aberrant methylation. J Cancer. 2017;8:1187–96. 28607593 10.7150/jca.18171 PMC5463433

[108] Murai M, Toyota M, Suzuki H, Satoh A, Sasaki Y, Akino K, et al. Aberrant methylation and silencing of the BNIP3 gene in colorectal and gastric cancer. Clin Cancer Res. 2005;11:1021–7. 15709167

[109] Calvisi DF, Ladu S, Gorden A, Farina M, Lee JS, Conner EA, et al. Mechanistic and prognostic significance of aberrant methylation in the molecular pathogenesis of human hepatocellular carcinoma. J Clin Investig. 2007;117:2713–22. 17717605 10.1172/JCI31457 PMC1950459

[110] Sun JL, He XS, Yu YH, Chen ZC. Expression and structure of BNIP3L in lung cancer. Chin J Cancer. 2004;23:8–14. 14720367

[111] Han H, Bearss DJ, Browne LW, Calaluce R, Nagle RB, Von Hoff DD. Identification of differentially expressed genes in pancreatic cancer cells using cDNA microarray. Cancer Res. 2002;62:2890–6. 12019169

[112] Okami J, Simeone DM, Logsdon CD. Silencing of the hypoxia-inducible cell death protein BNIP3 in pancreatic cancer. Cancer Res. 2004;64:5338–46. 15289340 10.1158/0008-5472.CAN-04-0089

[113] Fan S, Liu H, Hou J, Zheng G, Gu P, Liu X. Characterizing adipocytokine-related signatures for prognosis prediction in prostate cancer. Front Cell Dev Biol. 2024;12:1475980. 39524226 10.3389/fcell.2024.1475980 PMC11544632

[114] Lu Y, Wang L, He M, Huang W, Li H, Wang Y, et al. Nix protein positively regulates NF-κB activation in gliomas. PLoS One. 2012;7:e44559. 22984526 10.1371/journal.pone.0044559 PMC3440440

[115] Jung J, Zhang Y, Celiku O, Zhang W, Song H, Williams BJ, et al. Mitochondrial NIX promotes tumor survival in the hypoxic niche of glioblastoma. Cancer Res. 2019;79:5218–32. 31488423 10.1158/0008-5472.CAN-19-0198 PMC6801103

[116] Yang NX. Prognostic value and immunoregulatory mechanism of BNIP3L/Nix in breast cancer. BIO Web Conf. 2024;124:02018.

[117] Sowter HM, Ferguson M, Pym C, Watson P, Fox SB, Han C, et al. Expression of the cell death genes BNip3 and NIX in ductal carcinoma in situ of the breast; correlation of BNip3 levels with necrosis and grade. J Pathol. 2003;201:573–80. 14648660 10.1002/path.1486

[118] Yan C, Luo L, Guo CY, Goto S, Urata Y, Shao JH, et al. Doxorubicin-induced mitophagy contributes to drug resistance in cancer stem cells from HCT8 human colorectal cancer cells. Cancer Lett. 2017;388:34–42. 27913197 10.1016/j.canlet.2016.11.018

[119] Chen YY, Wang WH, Che L, Lan Y, Zhang LY, Zhan DL, et al. BNIP3L-dependent mitophagy promotes HBx-induced cancer stemness of hepatocellular carcinoma cells via glycolysis metabolism reprogramming. Cancers. 2020;12:655. 32168902 10.3390/cancers12030655 PMC7139741

[120] Sun L, Li T, Wei Q, Zhang Y, Jia X, Wan Z, et al. Upregulation of BNIP3 mediated by ERK/HIF-1α pathway induces autophagy and contributes to anoikis resistance of hepatocellular carcinoma cells. Future Oncol. 2014;10:1387–98. 25052749 10.2217/fon.14.70

[121] Uberall I, Kolek V, Klein J, Krejcí V, Stastná J, Radová L, et al. The immunohistochemical expression of BNIP3 protein in non-small-cell lung cancer: a tissue microarray study. APMIS. 2010;118:565–70. 20666737 10.1111/j.1600-0463.2010.02616.x

[122] Kazimierczak U, Kolenda T, Kowalczyk D, Mackiewicz J, Mackiewicz A. BNIP3L is a new autophagy- related prognostic biomarker for melanoma patients treated with AGI-101H. Anticancer Res. 2020;40:3723–32. 32620611 10.21873/anticanres.14361

[123] Watanabe R, Miura N, Kurata M, Kitazawa R, Kikugawa T, Saika T. Unveiling the genomic landscape of intraductal carcinoma of the prostate using spatial gene expression analysis. Int J Mol Sci. 2024;25:4818. 38732035 10.3390/ijms25094818 PMC11083946

[124] Nollet EA, Cardo-Vila M, Ganguly SS, Tran JD, Schulz VV, Cress A, et al. Androgen receptor-induced integrin α6β1 and Bnip3 promote survival and resistance to PI3K inhibitors in castration-resistant prostate cancer. Oncogene. 2020;39:5390–404. 32565538 10.1038/s41388-020-1370-9 PMC7395876

[125] Kim JH, Kim HS, Lee S. BCL2L13 protein prevents apoptosis in acute myeloid leukemia cells. Oncologie. 2023;25:345–54.

[126] Tandel P, Ranjbaran R, Ebrahimi E, Rezvani A, Ramzi M, Tamaddon G. Decreased expression of autophagy-related genes in the complete remission phase of acute myeloid leukemia. Mol Genet Genom Med. 2022;10:e1872. 10.1002/mgg3.1872 PMC8922948 35128828

[127] Wang J, Chen A, Xue Z, Liu J, He Y, Liu G, et al. BCL2L13 promotes mitophagy through DNM1L-mediated mitochondrial fission in glioblastoma. Cell Death Dis. 2023;14:585. 37660127 10.1038/s41419-023-06112-4 PMC10475114

[128] Dowling AL, Walbridge S, Ertekin C, Namagiri S, Camacho K, Chowdhury A, et al. FKBP38 regulates self-renewal and survival of GBM neurospheres. Cells. 2023;12:2562. 37947640 10.3390/cells12212562 PMC10647221

[129] Falasca L, Torino F, Marconi M, Costantini M, Pompeo V, Sentinelli S, et al. AMBRA1 and SQSTM1 expression pattern in prostate cancer. Apoptosis. 2015;20:1577–86. 26423274 10.1007/s10495-015-1176-3 PMC4602066

[130] Yu G, Chen S, Chen X, Hou K, Liang M. miR-222 enhances HBx-HepG2 cell growth via regulation of BCL2L13 gene. Chin J Pathophysiol. 2016;32:1389–94.

[131] Chaikovsky AC, Li C, Jeng EE, Loebell S, Lee MC, Murray CW, et al. The AMBRA1 E3 ligase adaptor regulates the stability of cyclin D. Nature. 2021;592:794–8. 33854239 10.1038/s41586-021-03474-7 PMC8246597

[132] Fong S, Mounkes L, Liu Y, Maibaum M, Alonzo E, Desprez PY, et al. Functional identification of distinct sets of antitumor activities mediated by the FKBP gene family. Proc Natl Acad Sci USA. 2003;100:14253–8. 14612567 10.1073/pnas.2332307100 PMC283578

[133] Tang DYL, Ellis RA, Lovat PE. Prognostic impact of autophagy biomarkers for cutaneous melanoma. Front Oncol. 2016;6:236. 27882308 10.3389/fonc.2016.00236 PMC5101199

[134] Wu L, Zhang D, Zhou L, Pei Y, Zhuang Y, Cui W, et al. FUN14 domain-containing 1 promotes breast cancer proliferation and migration by activating the calcium-NFATC1-BMI1 axis. EBioMedicine. 2019;41:384–94. 30803933 10.1016/j.ebiom.2019.02.032 PMC6442990

[135] Yuan Q, Sun N, Zheng J, Wang Y, Yan X, Mai W, et al. Prognostic and immunological role of FUN14 domain-containing 1 in Pan-cancer: friend or foe? Front Oncol. 2020;9:1502. 31998650 10.3389/fonc.2019.01502 PMC6966411

[136] Ren L, Meng L, Gao J, Lu M, Guo C, Li Y, et al. PHB2 promotes colorectal cancer cell proliferation and tumorigenesis through NDUFS1-mediated oxidative phosphorylation. Cell Death Dis. 2023;14:44. 36658121 10.1038/s41419-023-05575-9 PMC9852476

[137] Cheng J, Gao F, Chen X, Wu J, Xing C, Lv Z, et al. Prohibitin-2 promotes hepatocellular carcinoma malignancy progression in hypoxia based on a label-free quantitative proteomics strategy. Mol Carcinog. 2014;53:820–32. 23661548 10.1002/mc.22040

[138] Wu B, Chang N, Xi H, Xiong J, Zhou Y, Wu Y, et al. PHB2 promotes tumorigenesis via RACK1 in non-small cell lung cancer. Theranostics. 2021;11:3150–66. 33537079 10.7150/thno.52848 PMC7847695

[139] Najem A, Krayem M, Sabbah S, Pesetti M, Journe F, Awada A, et al. Targeting prohibitins to inhibit melanoma growth and overcome resistance to targeted therapies. Cells. 2023;12:1855. 37508519 10.3390/cells12141855 PMC10378173

[140] Dimitrijevs P, Freiliba I, Pčolkins A, Leja M, Arsenyan P. Total cardiolipin levels in gastric and colon cancer: evaluating the prognostic potential. Lipids Health Dis. 2025;24:76. 40016755 10.1186/s12944-025-02499-5 PMC11866619

[141] Zhong H, Xiao M, Zarkovic K, Zhu M, Sa R, Lu J, et al. Mitochondrial control of apoptosis through modulation of cardiolipin oxidation in hepatocellular carcinoma: A novel link between oxidative stress and cancer. Free Radic Biol Med. 2017;102:67–76. 27838437 10.1016/j.freeradbiomed.2016.10.494

[142] Xue X, Tan H, Jiang X, Lu J, Sun T, Yang W. Prohibitin2 knockdown decreases glioma malignant phenotypes and radio-resistance by inhibiting mitophagy. Int J Radiat Biol. 2025;101:487–98. 40029335 10.1080/09553002.2025.2470203

[143] Rodrigo R, Mendis N, Ibrahim M, Ma C, Kreinin E, Roma A, et al. Knockdown of BNIP3L or SQSTM1 alters cellular response to mitochondria-targeted drugs. Autophagy. 2019;15:900–7. 30563411 10.1080/15548627.2018.1558002 PMC6526872

[144] Salwa A, Ferraresi A, Vallino L, Maheshwari C, Moia R, Gaidano G, et al. High mitophagy and low glycolysis predict better clinical outcomes in acute myeloid leukemias. Int J Mol Sci. 2024;25:11527. 39519080 10.3390/ijms252111527 PMC11546612

[145] Lee YE, Lim HJ, Park JH, Kim HR, Kang MG, Cho YK, et al. Overexpression of prohibitin 2 protein is associated with adverse prognosis in cytogenetically normal acute myeloid leukemia. Ann Lab Med. 2022;42:585–9. 35470276 10.3343/alm.2022.42.5.585 PMC9057826

[146] Burton TR, Henson ES, Baijal P, Eisenstat DD, Gibson SB. The pro-cell death Bcl-2 family member, BNIP3, is localized to the nucleus of human glial cells: Implications for glioblastoma multiforme tumor cell survival under hypoxia. Int J Cancer. 2006;118:1660–9. 16217754 10.1002/ijc.21547 PMC3158801

[147] Zhang Y, Li W, Yang Y, Zhang S, Yang H, Hao Y, et al. AAA237, an SKP2 inhibitor, suppresses glioblastoma by inducing BNIP3-dependent autophagy through the mTOR pathway. Cancer Cell Int. 2024;24:69. 38341584 10.1186/s12935-023-03191-3 PMC10859026

[148] Buratta M, Castigli E, Sciaccaluga M, Pellegrino RM, Spinozzi F, Roberti R, et al. Loss of cardiolipin in palmitate-treated GL15 glioblastoma cells favors cytochrome c release from mitochondria, leading to apoptosis. J Neurochem. 2008;105:1019–31. 18182042 10.1111/j.1471-4159.2007.05209.x

[149] Petry IB, Fieber E, Schmidt M, Gehrmann M, Gebhard S, Hermes M, et al. ERBB2 induces an antiapoptotic expression pattern of Bcl-2 family members in node-negative breast cancer. Clin Cancer Res. 2010;16:451–60. 20068093 10.1158/1078-0432.CCR-09-1617

[150] Liu J, Yuan B, Cao J, Luo H, Gu S, Zhang M, et al. AMBRA1 promotes TGFβ signaling via nonproteolytic polyubiquitylation of Smad4. Cancer Res. 2021;81:5007–20. 34362797 10.1158/0008-5472.CAN-21-0431

[151] Taniguchi K, Matsumura K, Kageyama S, Ii H, Ashihara E, Chano T, et al. Prohibitin-2 is a novel regulator of p21WAF1/CIP1 induced by depletion of γ-glutamylcyclotransferase. Biochem Biophys Res Commun. 2018;496:218–24. 29307834 10.1016/j.bbrc.2018.01.029

[152] Wang Y, Han C, Lu L, Magliato S, Wu T. Hedgehog signaling pathway regulates autophagy in human hepatocellular carcinoma cells. Hepatology. 2013;58:995–1010. 23504944 10.1002/hep.26394 PMC3706478

[153] Berardi DE, Bock-Hughes A, Terry AR, Drake LE, Bozek G, Macleod KF. Lipid droplet turnover at the lysosome inhibits growth of hepatocellular carcinoma in a BNIP3-dependent manner. Sci Adv. 2022;8:eabo2510. 36223464 10.1126/sciadv.abo2510 PMC9555787

[154] Li W, Li Y, Siraj S, Jin H, Fan Y, Yang X, et al. FUN14 domain-containing 1-mediated mitophagy suppresses hepatocarcinogenesis by inhibition of inflammasome activation in mice. Hepatology. 2019;69:604–21. 30053328 10.1002/hep.30191

[155] Lee HY, Oh SH. Arsenite-induced cytotoxicity is regulated by p38-SQSTM1/p62 and JNK-BNIP3L/Nix signaling in lung cancer cells. Biochem Biophys Res Commun. 2022;587:16–23. 34861471 10.1016/j.bbrc.2021.11.068

[156] Di Leo L, Bodemeyer V, Bosisio FM, Claps G, Carretta M, Rizza S, et al. Loss of Ambra1 promotes melanoma growth and invasion. Nat Commun. 2021;12:2550. 33953176 10.1038/s41467-021-22772-2 PMC8100102

[157] Humpton TJ, Alagesan B, DeNicola GM, Lu D, Yordanov GN, Leonhardt CS, et al. Oncogenic KRAS induces NIX-mediated mitophagy to promote pancreatic cancer. Cancer Discov. 2019;9:1268–87. 31263025 10.1158/2159-8290.CD-18-1409 PMC6726540

[158] Masuo H, Kubota K, Shimizu A, Notake T, Miyazaki S, Yoshizawa T, et al. Increased mitochondria are responsible for the acquisition of gemcitabine resistance in pancreatic cancer cell lines. Cancer Sci. 2023;114:4388–4400. 37700464 10.1111/cas.15962 PMC10637055

[159] Wen C, Ge Q, Dai B, Li J, Yang F, Meng J, et al. Signature for prostate cancer based on autophagy-related genes and a nomogram for quantitative risk stratification. Dis Markers. 2022;2022:7598942. 10.1155/2022/7598942 PMC9293571 35860692

[160] Huang Y, Shen P, Chen X, Chen Z, Zhao T, Chen N, et al. Transcriptional regulation of BNIP3 by Sp3 in prostate cancer. Prostate. 2015;75:1556–67. 26012884 10.1002/pros.23029

[161] Shen Y, Gao Y, Yuan H, Cao J, Jia B, Li M, et al. Prohibitin-2 negatively regulates AKT2 expression to promote prostate cancer cell migration. Int J Mol Med. 2018;41:1147–55. 29207197 10.3892/ijmm.2017.3307

[162] Saklayen MG. The global epidemic of the metabolic syndrome. Curr Hypertens Rep. 2018;20:12. 29480368 10.1007/s11906-018-0812-z PMC5866840

[163] Su Z, Nie Y, Huang X, Zhu Y, Feng B, Tang L, et al. Mitophagy in hepatic insulin resistance: therapeutic potential and concerns. Front Pharmacol. 2019;10:1193. 31649547 10.3389/fphar.2019.01193 PMC6795753

[164] Levi-D’Ancona E, Stendahl AM, Henry-Kanarek BA, Davidson RK, Walker EM, Soleimanpour SA. Mitophagy in the adaptation to pancreatic β-cell stress in diabetes. Trends Endocrinol Metab 2025. 10.1016/j.tem.2025.09.009 41109799

[165] Sidarala V, Pearson GL, Parekh VS, Thompson B, Christen L, Gingerich MA, et al. Mitophagy protects β cells from inflammatory damage in diabetes. JCI Insight. 2020;5:e141138. 33232298 10.1172/jci.insight.141138 PMC7819751

[166] Fujimoto K, Ford EL, Tran H, Wice BM, Crosby SD, Dorn GW, et al. Loss of Nix in Pdx1-deficient mice prevents apoptotic and necrotic β-cell death and diabetes. J Clin Invest. 2010;120:4031–9. 20978346 10.1172/JCI44011 PMC2964996

[167] Supale S, Thorel F, Merkwirth C, Gjinovci A, Herrera PL, Scorrano L, et al. Loss of prohibitin induces mitochondrial damage, altering β-cell function and survival and is responsible for gradual diabetes development. Diabetes. 2013;62:3488–99. 23863811 10.2337/db13-0152 PMC3781460

[168] Da Silva Rosa SC, Martens MD, Field JT, Nguyen L, Kereliuk SM, Hai Y, et al. BNIP3L/Nix-induced mitochondrial fission, mitophagy, and impaired myocyte glucose uptake are abrogated by PRKA/PKA phosphorylation. Autophagy. 2021;17:2257–72. 33044904 10.1080/15548627.2020.1821548 PMC8496715

[169] Tong B, Zhang Z, Li X, Liu J, Wang H, Song L, et al. FUNDC1 modulates mitochondrial defects and pancreatic β-cell dysfunction under lipotoxicity. Biochem Biophys Res Commun. 2023;672:54–64. 37336125 10.1016/j.bbrc.2023.06.042

[170] Wu H, Wang Y, Li W, Chen H, Du L, Liu D, et al. Deficiency of mitophagy receptor FUNDC1 impairs mitochondrial quality and aggravates diet-induced obesity and metabolic syndrome. Autophagy. 2019;15:1882–98. 30898010 10.1080/15548627.2019.1596482 PMC6844496

[171] Grepper D, Tabasso C, Zanou N, Aguettaz AKF, Castro-Sepulveda M, Ziegler DV, et al. BCL2L13 at endoplasmic reticulum-mitochondria contact sites regulates calcium homeostasis to maintain skeletal muscle function. iScience. 2024;27:110510. 39175772 10.1016/j.isci.2024.110510 PMC11340602

[172] Coccurello R, Nazio F, Rossi C, De Angelis F, Vacca V, Giacovazzo G, et al. Effects of caloric restriction on neuropathic pain, peripheral nerve degeneration and inflammation in normometabolic and autophagy-defective prediabetic Ambra1 mice. PLoS One. 2018;13:e0208596. 30532260 10.1371/journal.pone.0208596 PMC6287902

[173] El-Hafidi M, Correa F, Zazueta C. Mitochondrial dysfunction in metabolic and cardiovascular diseases associated with cardiolipin remodeling. Biochim Biophys Acta Mol Basis Dis. 2020;1866:165744. 32105822 10.1016/j.bbadis.2020.165744

[174] Yamauchi S, Sugiura Y, Yamaguchi J, Zhou X, Takenaka S, Odawara T, et al. Mitochondrial fatty acid oxidation drives senescence. Sci Adv. 2024;10:eado5887. 39454000 10.1126/sciadv.ado5887 PMC11506141

[175] Cha HJ. Erythropoiesis: insights from a genomic perspective. Exp Mol Med. 2024;56:2099–104. 39349824 10.1038/s12276-024-01311-1 PMC11542026

[176] Diwan A, Koesters AG, Odley AM, Pushkaran S, Baines CP, Spike BT, et al. Unrestrained erythroblast development in Nix-/- mice reveals a mechanism for apoptotic modulation of erythropoiesis. Proc Natl Acad Sci USA. 2007;104:6794–9. 17420462 10.1073/pnas.0610666104 PMC1849960

[177] Schweers RL, Zhang J, Randall MS, Loyd MR, Li W, Dorsey FC, et al. NIX is required for programmed mitochondrial clearance during reticulocyte maturation. Proc Natl Acad Sci USA. 2007;104:19500–5. 18048346 10.1073/pnas.0708818104 PMC2148318

[178] Yang LY, Cui NB, Wang HQ, Fu R, Qu W, Ruan EB, et al. NIX-mediated mitochondrial autophagy in pathogenesis of myelodysplastic syndrome anemia. Zhonghua Yi Xue Za Zhi. 2017;97:1071–5. 28395431 10.3760/cma.j.issn.0376-2491.2017.14.009

[179] Liu X, Zhang Y, Ni M, Cao H, Signer RAJ, Li D, et al. Regulation of mitochondrial biogenesis in erythropoiesis by mTORC1-mediated protein translation. Nat Cell Biol. 2017;19:626–38. 28504707 10.1038/ncb3527 PMC5771482

[180] Ali S, Mumtaz S, Shakir HA, Khan M, Tahir HM, Mumtaz S, et al. Current status of beta-thalassemia and its treatment strategies. Mol Genet Genom Med. 2021;9:e1788. 10.1002/mgg3.1788 PMC8683628 34738740

[181] Long Y, Zhang Q, Ling L, Zhuang Y, Wei X, Huang H, et al. Mutations in AMBRA1 aggravate β-thalassemia by impairing autophagy-mediated clearance of free α-globin. Blood. 2025;145:1074–88. 39693613 10.1182/blood.2023022688

[182] Noulsri E, Lerdwana S. Increased BNIP3 activity contributes to mitochondrial dysregulation: a hypothesis on the mechanism of ineffective erythropoiesis in β-thalassemia. Med Hypotheses. 2024;189:111395.

[183] Geng G, Liu J, Xu C, Pei Y, Chen L, Mu C, et al. Receptor-mediated mitophagy regulates EPO production and protects against renal anemia. Elife. 2021;10:e64480. 33942716 10.7554/eLife.64480 PMC8121547

[184] Loftus EV, Sandborn WJ. Epidemiology of inflammatory bowel disease. Gastroenterol Clin North Am. 2002;31:1–20. 12122726 10.1016/s0889-8553(01)00002-4

[185] Vincent G, Novak EA, Siow VS, Cunningham KE, Griffith BD, Comerford TE, et al. Nix-mediated mitophagy modulates mitochondrial damage during intestinal inflammation. Antioxid Redox Signal. 2020;33:1–19. 32103677 10.1089/ars.2018.7702 PMC7262642

[186] Chen Y, Li J, Li S, Cheng Y, Fu X, Li J, et al. Uncovering the novel role of NR1D1 in regulating BNIP3-mediated mitophagy in ulcerative colitis. Int J Mol Sci. 2023;24:14222. 37762536 10.3390/ijms241814222 PMC10531686

[187] Xu W, Hua Z, Wang Y, Tang W, Ou W, Liu F, et al. AMBRA1 promotes intestinal inflammation by antagonizing PP4R1/PP4c-mediated IKK dephosphorylation in an autophagy-independent manner. Cell Death Differ. 2024;31:618–34. 38424148 10.1038/s41418-024-01275-9 PMC11094188

[188] Zuo L, Kuo WT, Cao F, Chanez-Paredes SD, Zeve D, Mannam P, et al. Tacrolimus-binding protein FKBP8 directs myosin light chain kinase-dependent barrier regulation and is a potential therapeutic target in Crohn’s disease. Gut. 2023;72:870–81. 35537812 10.1136/gutjnl-2021-326534 PMC9977574

[189] Lanier LL. NK cell recognition. Annu Rev Immunol. 2005;23:225–74. 15771571 10.1146/annurev.immunol.23.021704.115526

[190] O’Sullivan TE, Johnson LR, Kang HH, Sun JC. BNIP3- and BNIP3L-mediated mitophagy promote the generation of natural killer cell memory. Immunity. 2015;43:331–42. 26253785 10.1016/j.immuni.2015.07.012 PMC5737626

[191] Zhang W, Ma Q, Siraj S, Ney PA, Liu J, Liao X, et al. Nix-mediated mitophagy regulates platelet activation and life span. Blood Adv. 2019;3:2342–54. 31391167 10.1182/bloodadvances.2019032334 PMC6693007

